# Turning the Other Lobe: Directional Biases in Brain Diagrams

**DOI:** 10.1177/2041669517707769

**Published:** 2017-05-18

**Authors:** Richard Wiseman, Adrian M. Owen

**Affiliations:** Department of Psychology, University of Hertfordshire, Hatfield, Herts, UK; The Brain and Mind Institute, The University of Western Ontario, London, Ontario, Canada

**Keywords:** body perception, cognition, face perception, perception

## Abstract

Past research shows that in drawn or photographic portraits, people are significantly more likely to be posed facing to their right than their left. We examined whether the same type of bias exists among sagittal images of the human brain. An exhaustive search of Google images using the term ‘brain sagittal view’ yielded 425 images of a left or right facing brain. The direction of each image was coded and revealed that 80% of the brains were right-facing. This bias was present in images that did not contain any representation of a human head. It is argued that the effect might be aesthetic in nature, the result of the Western tradition of reading left to right or due to the facial factors that underlie the bias previously found in portraits.

Prior research has shown that people in both drawn and photographic portraits are more likely to be depicted looking to their right rather than their left (see, e.g., McManus & Humphrey, 1973; Gordon, 1974; Grusser, Selke & Zynda, 1988; Zaidel & Fitzgerald, 1994), and that this is probably due to the increased emotional expressiveness and perceived attractiveness of the left side of the face (Powell & Schirillo, 2009; Blackburn & Schirillo, 2012; Bruno & Bertamini, 2013; Bruno, Bertamini, & Protti, 2015).

Books, journal articles, and websites frequently carry diagrams of human brains, and we examined whether the same type of bias exists among these images. McManus (1979) notes that Dr Edwin Clarke pointed out the bias among 49 images of mediaeval drawings and woodcuts of the brain, and we wanted to discover whether the same bias exists in a much larger collection of more contemporary images. A Google image search using the term ‘brain sagittal view’ returned a total of 640 unique pictures, and 425 of these contained a human brain facing left or right. The direction of each brain was coded, along with whether it was embedded within a representation of a head or not (see Figure 1 and Supplemental Online Material). In line with previous research, 80% of the diagrams depicted a brain looking to its right (chi-squared = 82.67, *df* = 1, *p* < .01: Cramer’s V = 0.31: 95% CI [0.35, 0.51]).

**Figure 1.**
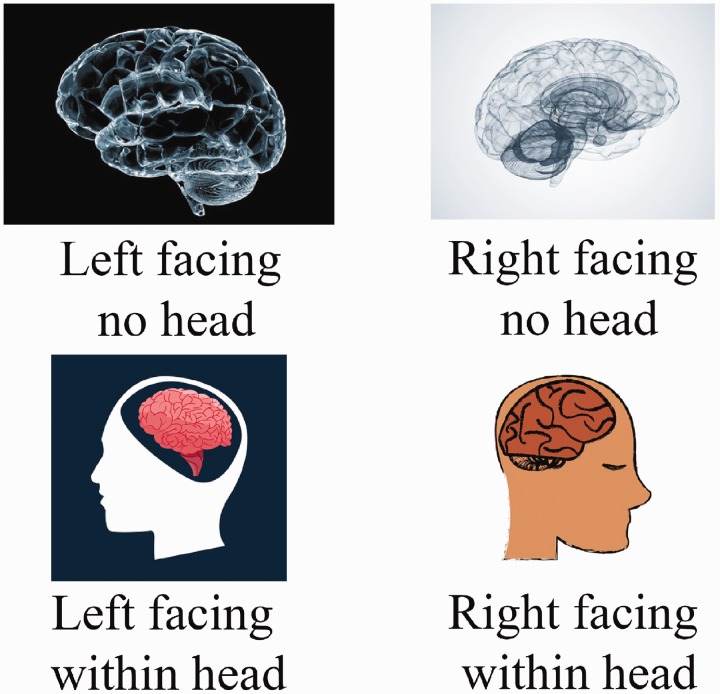
An illustration the ‘right’, ‘left’ and ‘head’, ‘no-head’ coding employed during the study.

Significantly, more brains were right facing in both the ‘no head’ (78.6%, chi-squared = 51.69, *df* = 1, *p* < .01: Cramer’s V = 0.30: 95% CI [0.31, 0.51]) and ‘head’ (83.1%, chi-Squared = 30.47, *df* = 1, *p* < .01: Cramer’s V = 0.35: 95% CI [0.32, 0.61]) conditions, and the difference between the conditions was not significant (chi-squared = .85, *p* = .36: *df* = 1, Cramer’s V = 0.05: 95% CI [−0.05, 0.31]), suggesting that the bias is not enhanced by the presence of head-related cues.

There is no structural or functional reason to depict the brain facing in one particular direction, and the bias may be due to several factors. For example, it could be the result of graphic artists finding it more aesthetically pleasing to have the brain facing to its right. Alternatively, because those in Western cultures read left to right, it makes more sense to encounter the anterior part of any image prior to the posterior (Suitner & McManus, 2011). Finally, it is possible that the effect might be, at least to some degree, due to seeing a greater proportion of portraits facing the right.

In addition, the same method was used to analyze roughly the same number of human profiles, and revealed additional evidence to support the notion that portraits of actual faces tend to be right facing (Google search term: ‘face side view’, *N* = 465, right facing 57.6%, chi-squared = 5.16, *p* = .02: *df* = 1, Cramer’s V = 0.08; 95% CI [0.25, 0.42]: see Supplemental Online Material).

In short, it is clear that in addition to turning the other cheek, many people are also turning the other lobe.

## Supplementary Material

Supplementary material
