# Corrigendum

**DOI:** 10.1177/2041669517716557

**Published:** 2017-06-14

Richard Wiseman, Adrian M. Owen. Turning the Other Lobe: Directional Biases in Brain Diagrams. i-Perception. 8(3):1–4.

The authors regret that the labels on Figure 1 are the wrong way round. They should be as given below:

**Figure 1.**
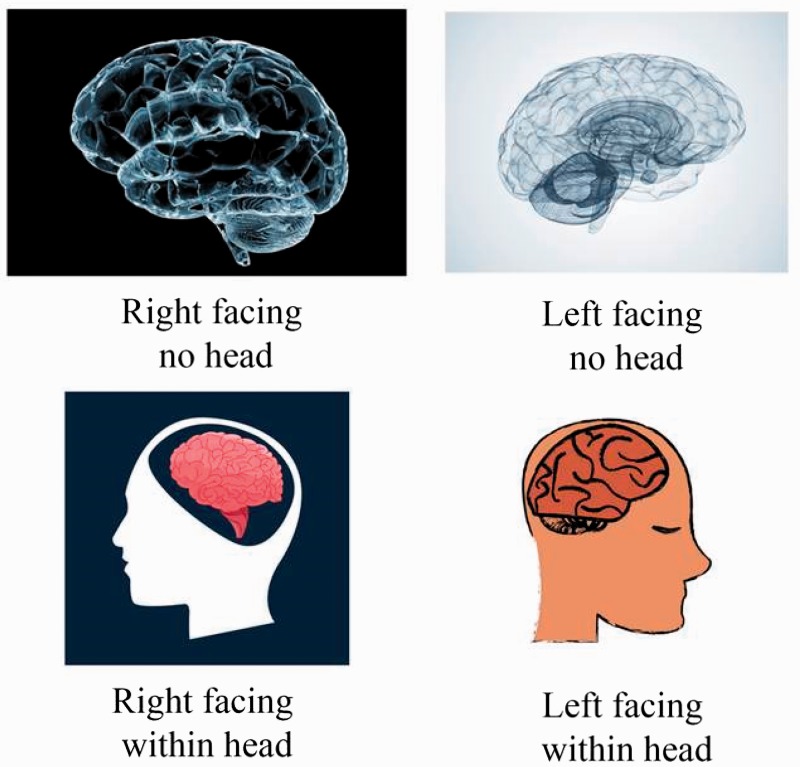
An illustration the ‘right’, ‘left’ and ‘head’, ‘no-head’ coding employed during the study.

