# Supplementary material for: Turning the Other Lobe: Directional Biases in Brain Diagrams
Source: Iperception. 2017 May 18;8(3):2041669517707769. doi: 10.1177/2041669517707769 (PMC5477996; doi:10.1177/2041669517707769)
Supplement: Supplementary material [file IPE707769_wisemanbrains.pdf]

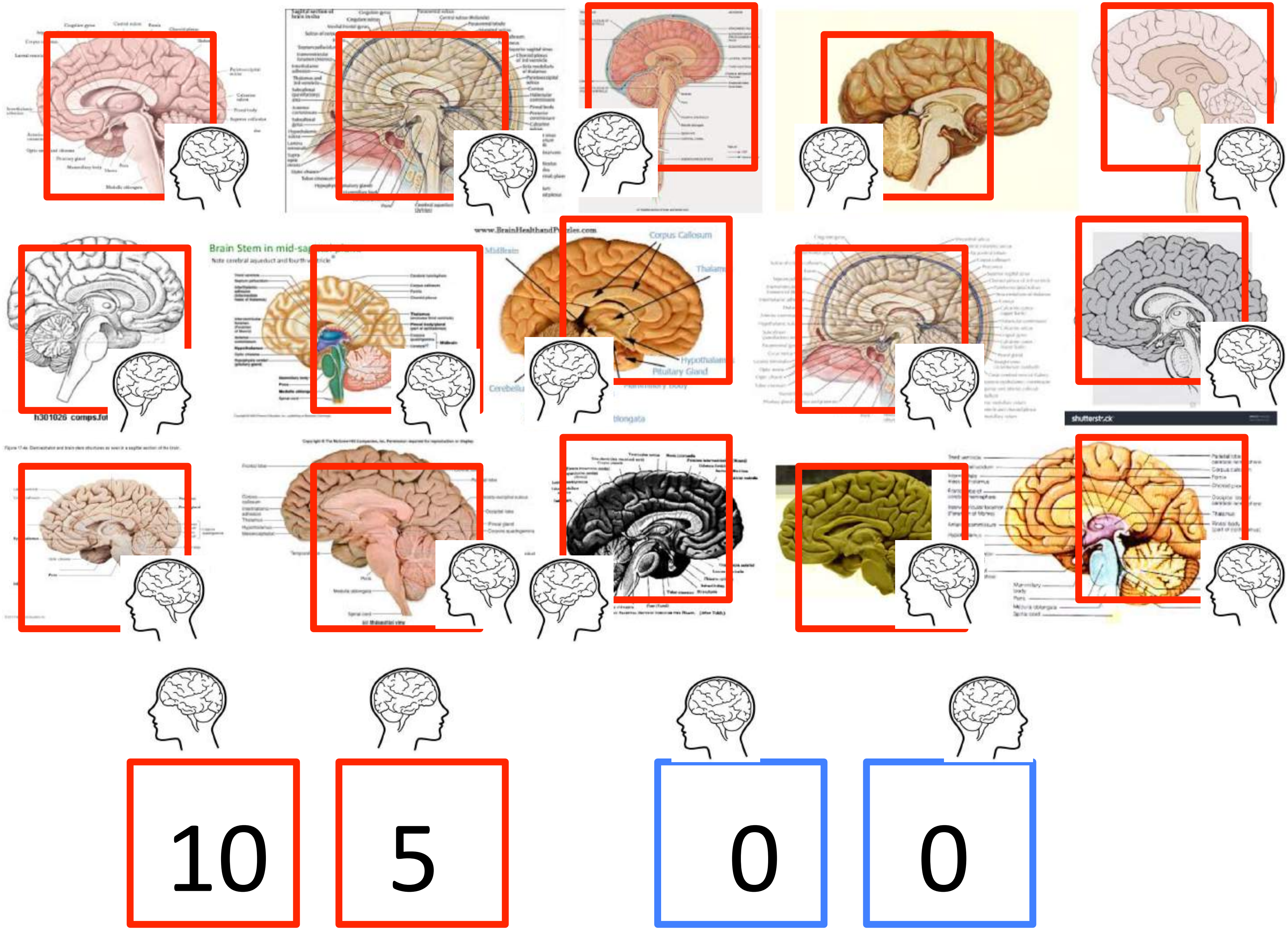

Corpus Callosum
Pituitary Gland
shutterstock
10
5
0
0

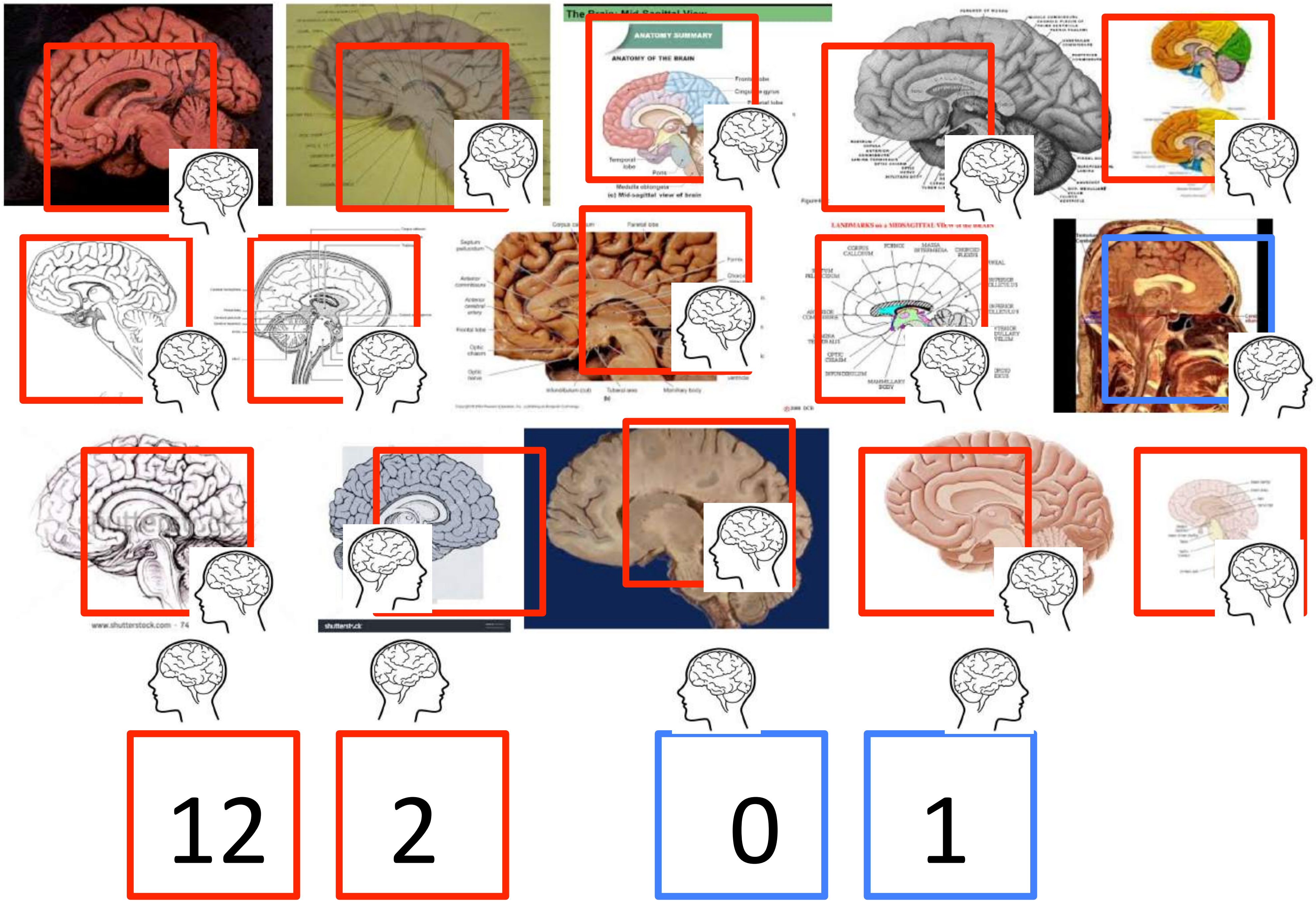
12
2
0
1

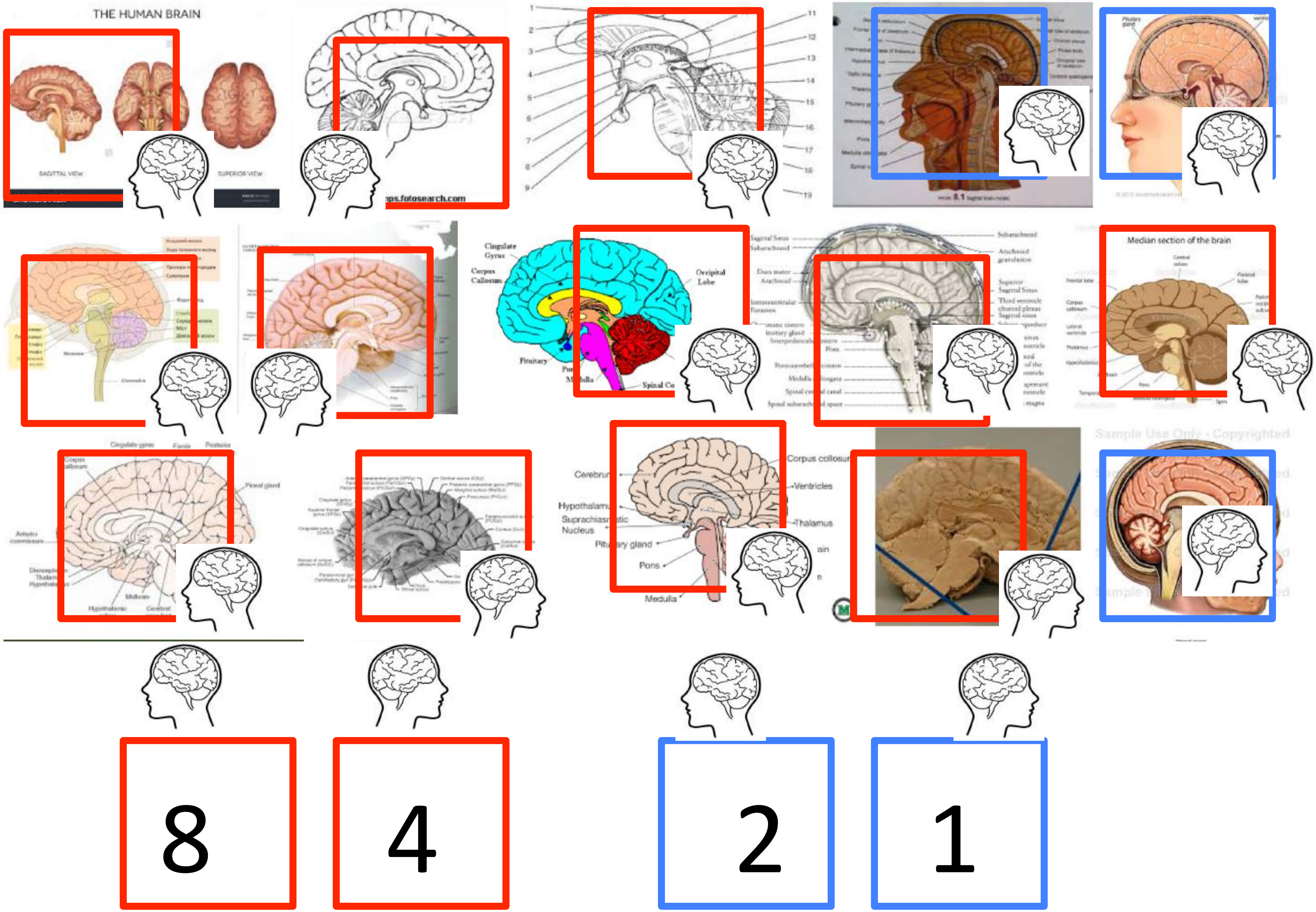
THE HUMAN BRAIN
SAGITTAL VIEW
SUPERIOR VIEW
Cingulate Gyrus
Corpus Callosum
Occipital Lobe
Pituitary
Median section of the brain
Ventricles
Thalamus
Pons
Medulla
Sample Use Only - Copyrighted
8
4
2
1

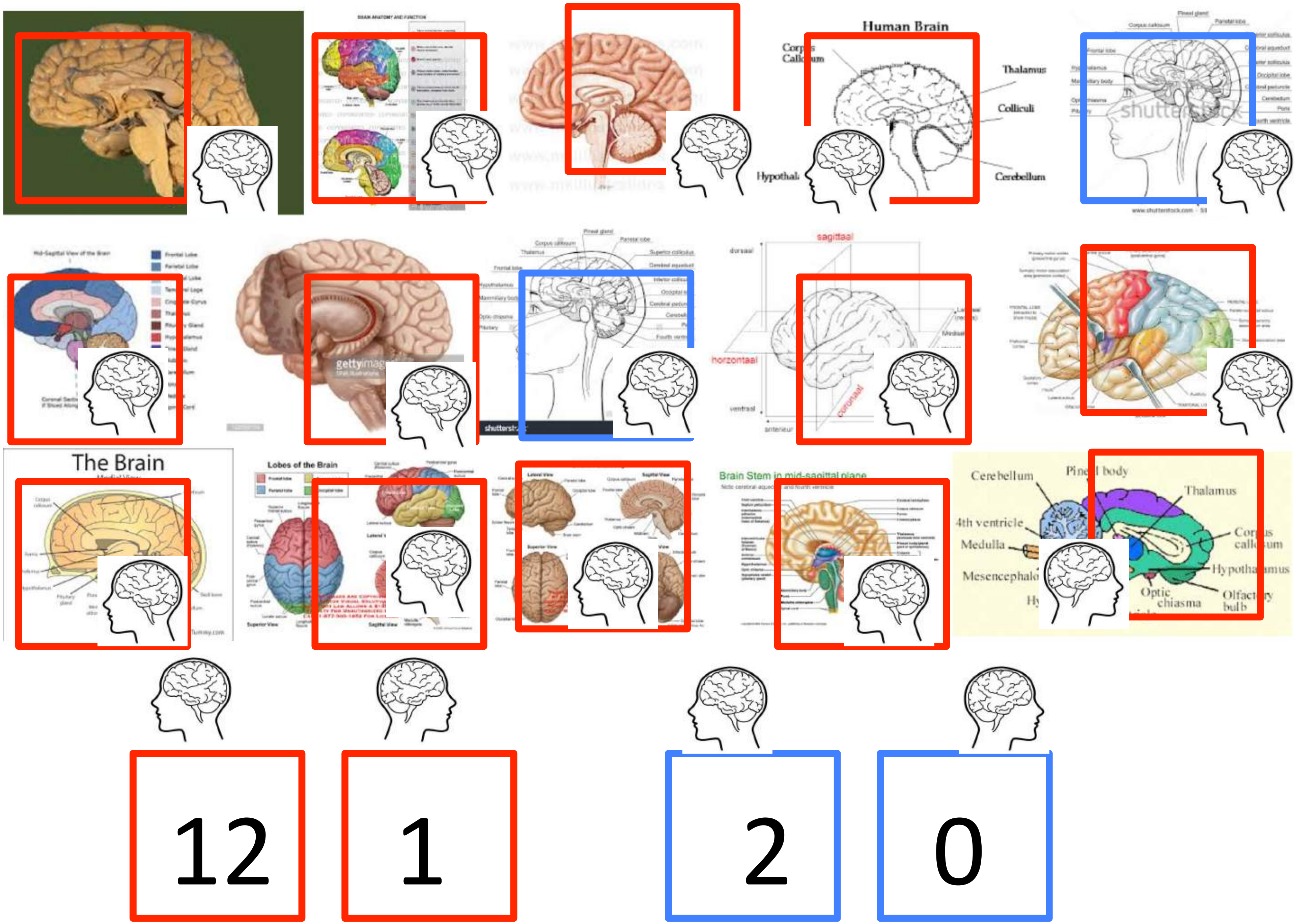
Human Brain
Thalamus
Colliculi
Cerebellum
The Brain
Lobes of the Brain
sagittaal
horzontaal
Brain Stem in mid-sagittal plane
Cerebellum
Thalamus
4th ventricle
Medulla
12
1
2
0

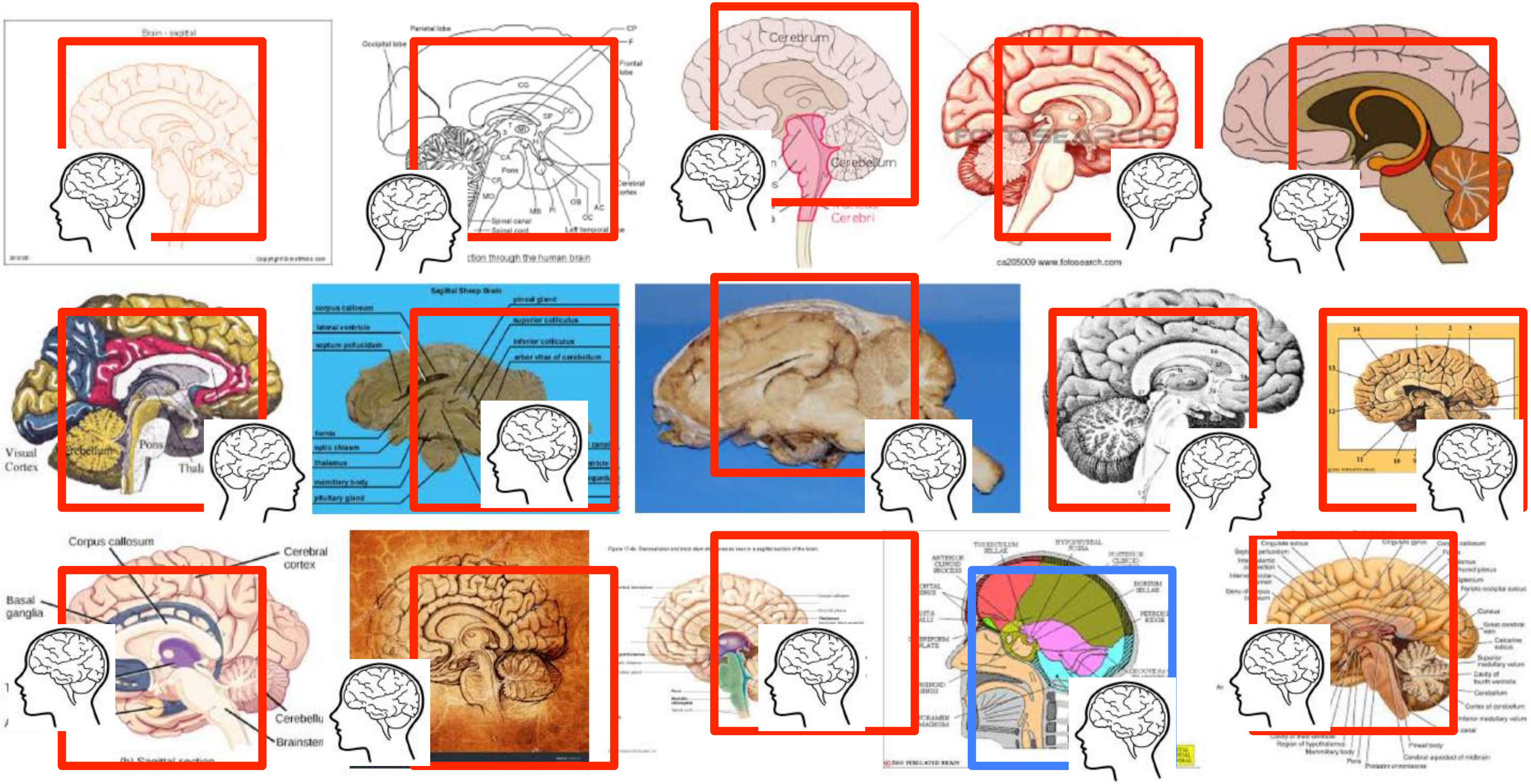
Cerebrum
Cerebellum
Corpus callosum
Cerebral cortex
Basal ganglia
Visual Cortex
Pons
Brainstem

10 | 4 | 1 | 0

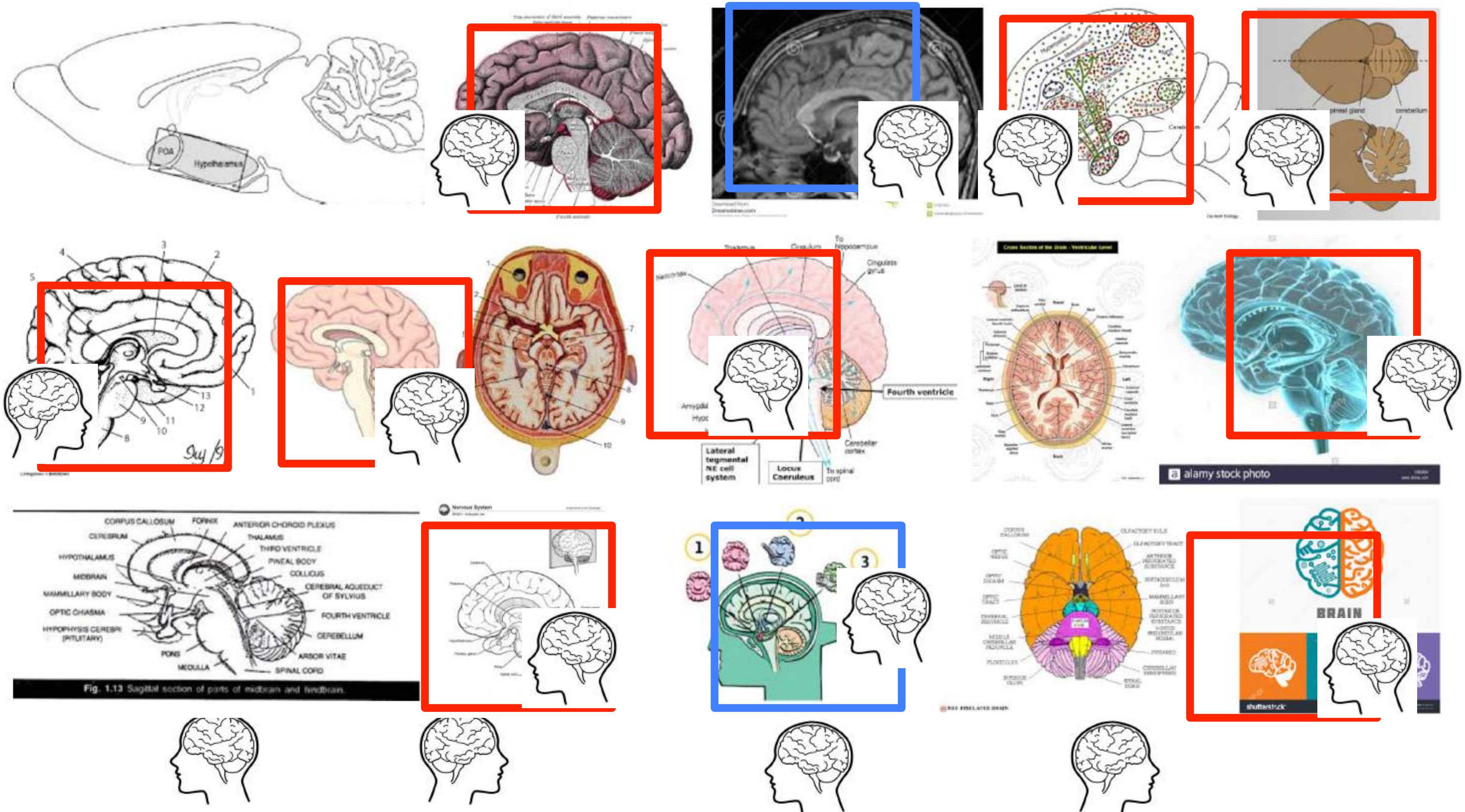
POA
Hypothalamus
Fourth ventricle
Lateral tegmental NE cell system
Locus Coeruleus
Cerebellar cortex
alamy stock photo
CORPUS CALLOSUM
FORNIX
ANTERIOR CHOROID PLEXUS
CEREBRUM
THALAMUS
THIRD VENTRICLE
HYPOTHALAMUS
PINEAL BODY
COLLICUS
MIDBRAIN
MAMMILLARY BODY
CEREBRAL AQUEDUCT OF SYLVIUS
OPTIC CHIASMA
FOURTH VENTRICLE
HYPOPHYSIS CEREBRI (PITUITARY)
CEREBELLUM
PONS
ARBOR VITAE
MEDULLA
SPINAL CORD
Fig. 1.13 Sagittal section of parts of midbrain and hindbrain
BRAIN
shutterstock

8 1 2 0

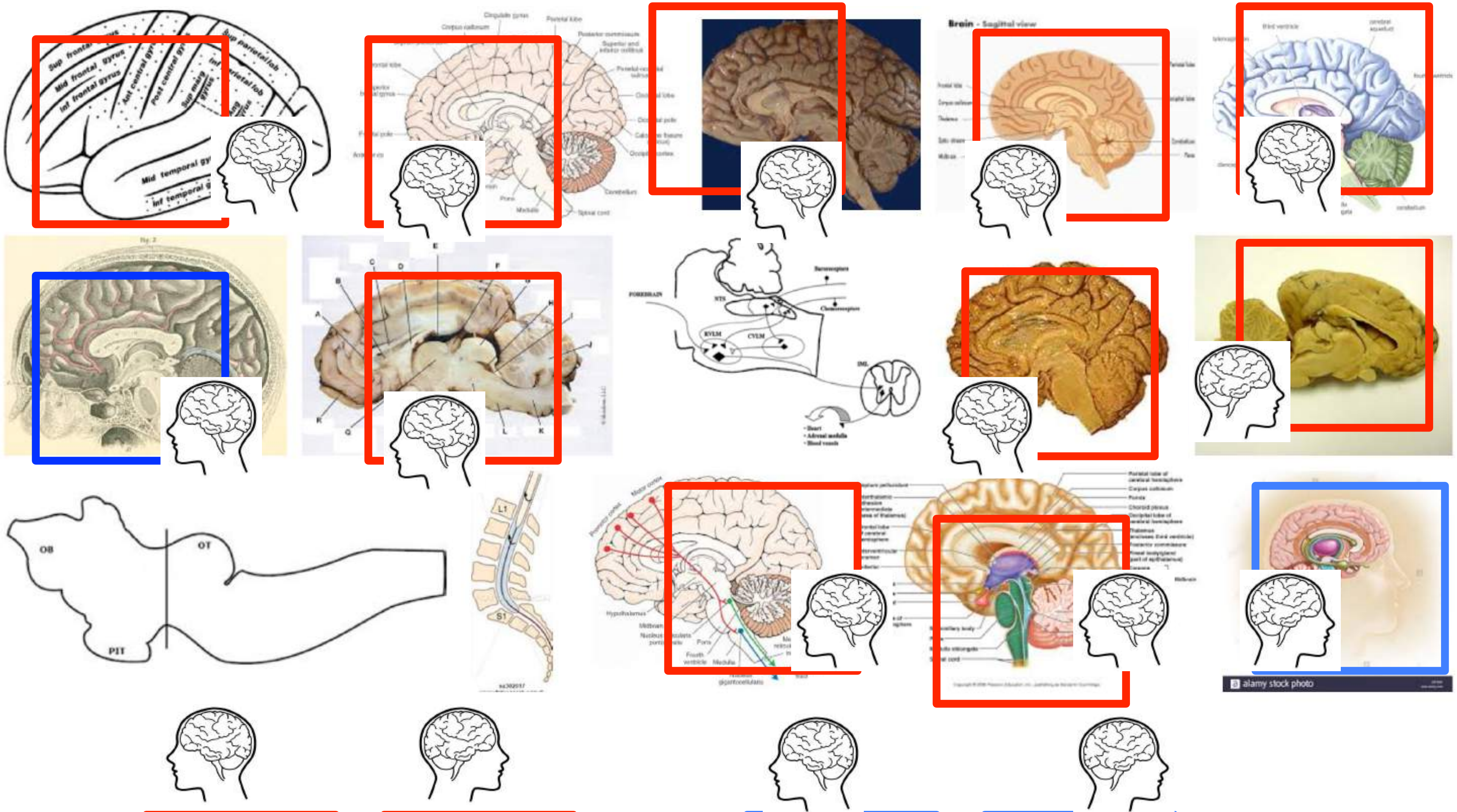

8

1

1

1

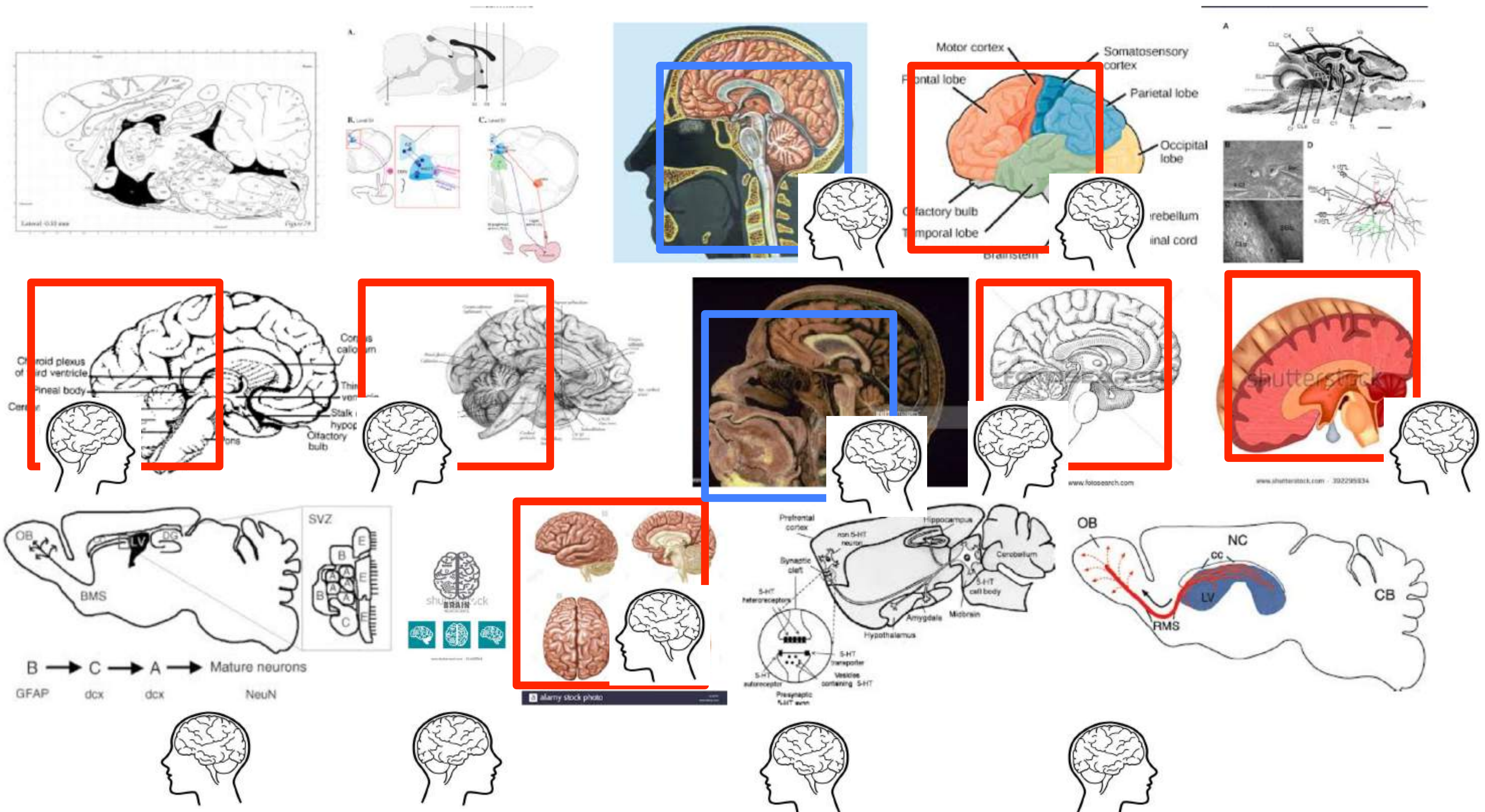

Motor cortex
Somatosensory cortex
Parietal lobe
Occipital lobe
Pineal body
Olfactory bulb
SVZ
OB
LV
DG
BMS
B → C → A → Mature neurons
GFAP
dcx
dcx
NeuN
alamy stock photo
Prefrontal cortex
Hippocampus
Cerebellum
Amygdala
Midbrain
Hypothalamus
OB
NC
CC
LV
RMS
CB

3

1

2

0

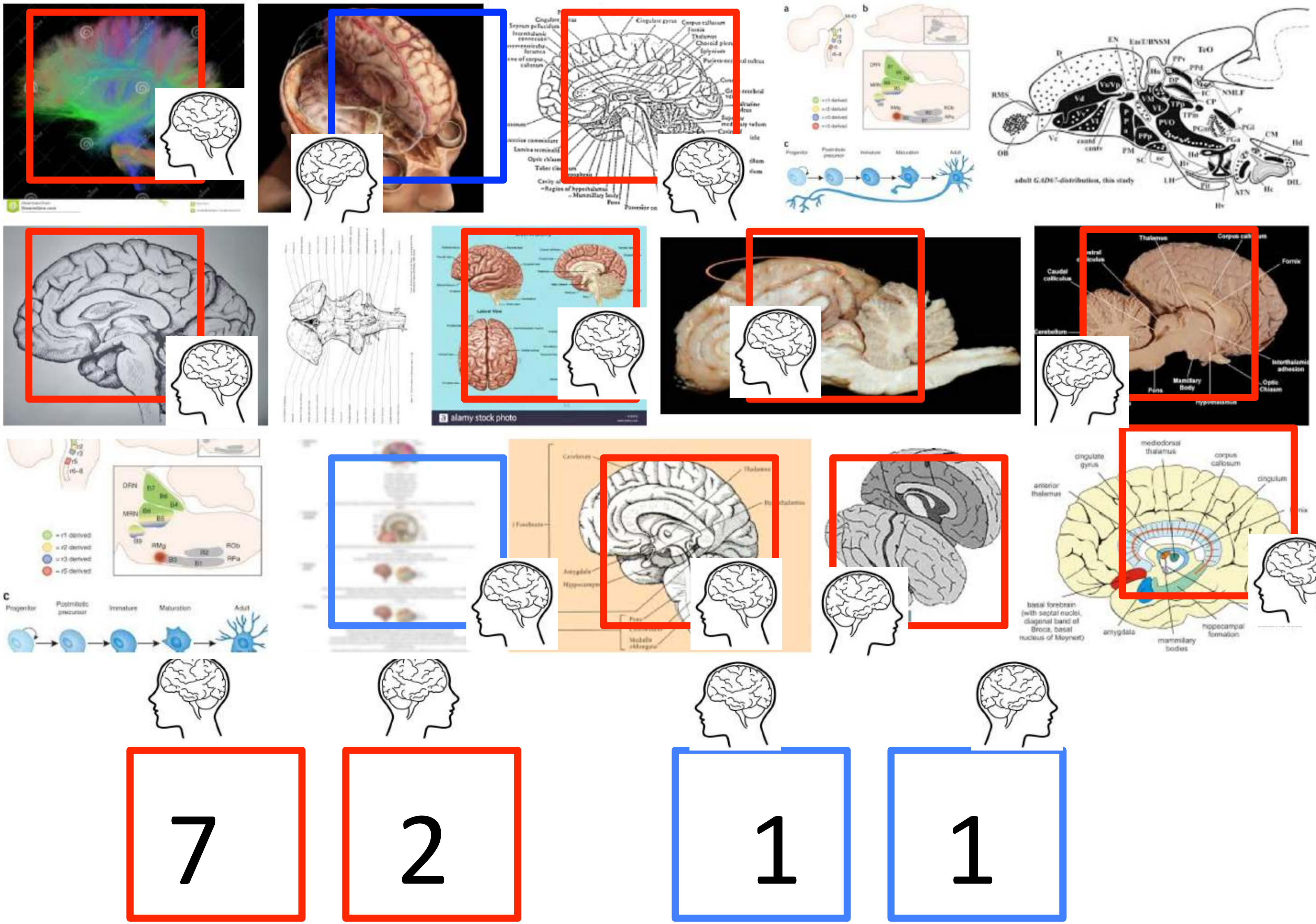

7
2
1
1

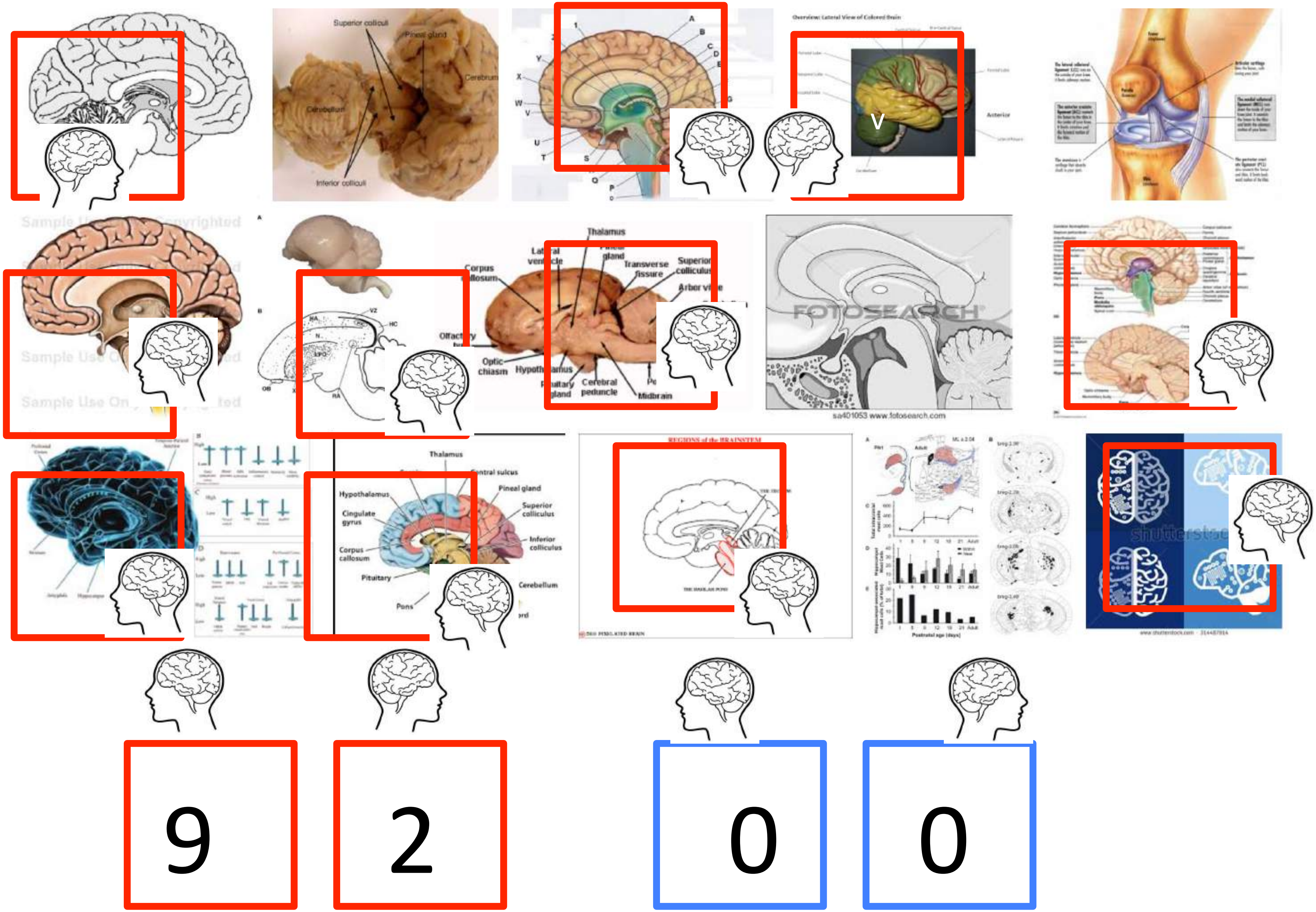
Superior colliculi
Pineal gland
Cerebrum
Cerebellum
Inferior colliculi
Thalamus
Lateral ventricle
Pineal gland
Corpus callosum
Transverse fissure
Superior colliculus
Arbor vitae
Optic chiasm
Cerebral peduncle
Midbrain
FOTOSEARCH
sa401053 www.fotosearch.com
Thalamus
Central sulcus
Hypothalamus
Pineal gland
Cingulate gyrus
Superior colliculus
Inferior colliculus
Corpus callosum
Pituitary
Cerebellum
Pons
REGIONS of the BRAINSTEM
shutterstock
9
2
0
0

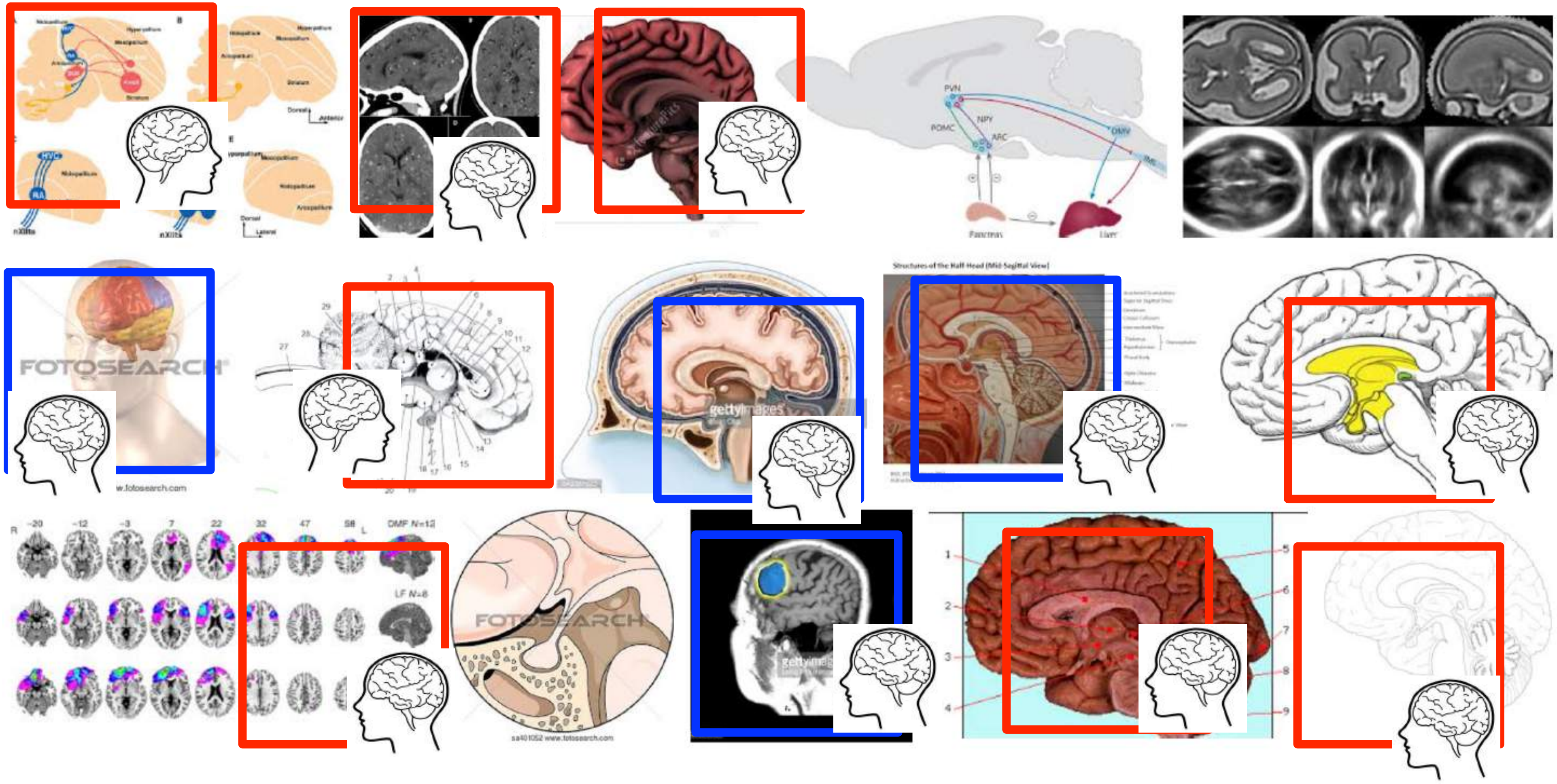

FOTOSEARCH
PVN
NPY
POMC
ARC
DMV
Pancreas
Liver
Structures of the Half-Head (Mid-Sagittal View)
gettyimages
DMF N=12
LF N=8
www.fotosearch.com

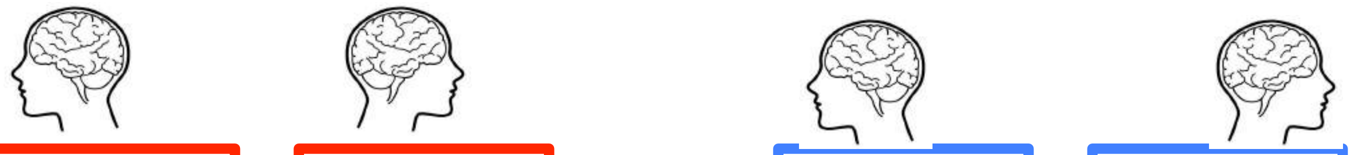

6	2	4	0

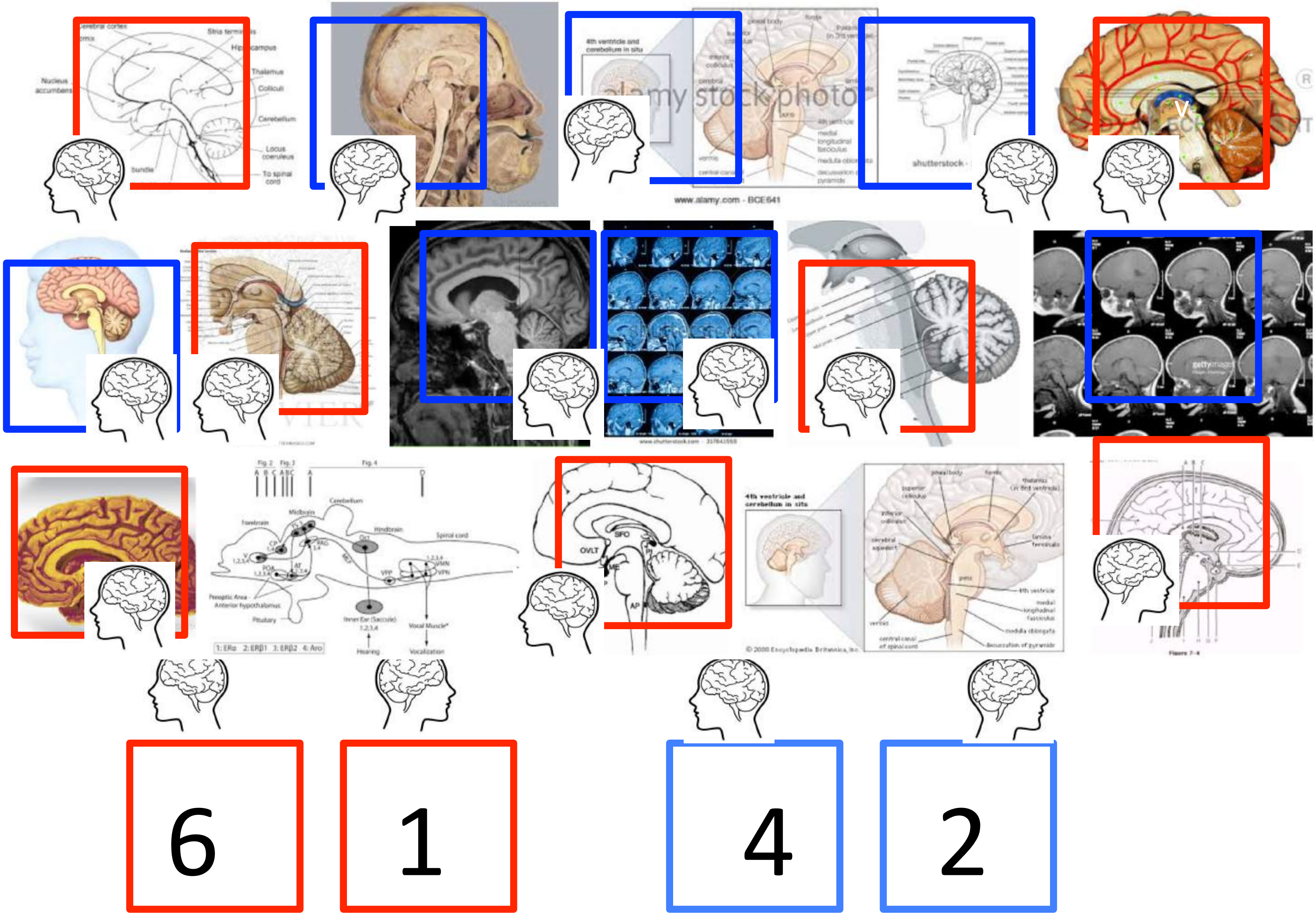
Nucleus accumbens
Thalamus
Colliculi
Cerebellum
Locus coeruleus
To spinal cord
4th ventricle and cerebellum in situ
alamy stock photo
www.alamy.com - BCE641
shutterstock
Forebrain
Midbrain
Cerebellum
Hindbrain
Spinal cord
Preoptic Area - Anterior hypothalamus
Pituitary
Inner Ear (Saccule)
Vocal Muscle
Hearing
Vocalization
1: ERα 2: ERβ1 3: ERβ2 4: Aro
OVLT
SFO
AP
4th ventricle and cerebellum in situ
© 2000 Encyclopaedia Britannica, Inc.
6
1
4
2

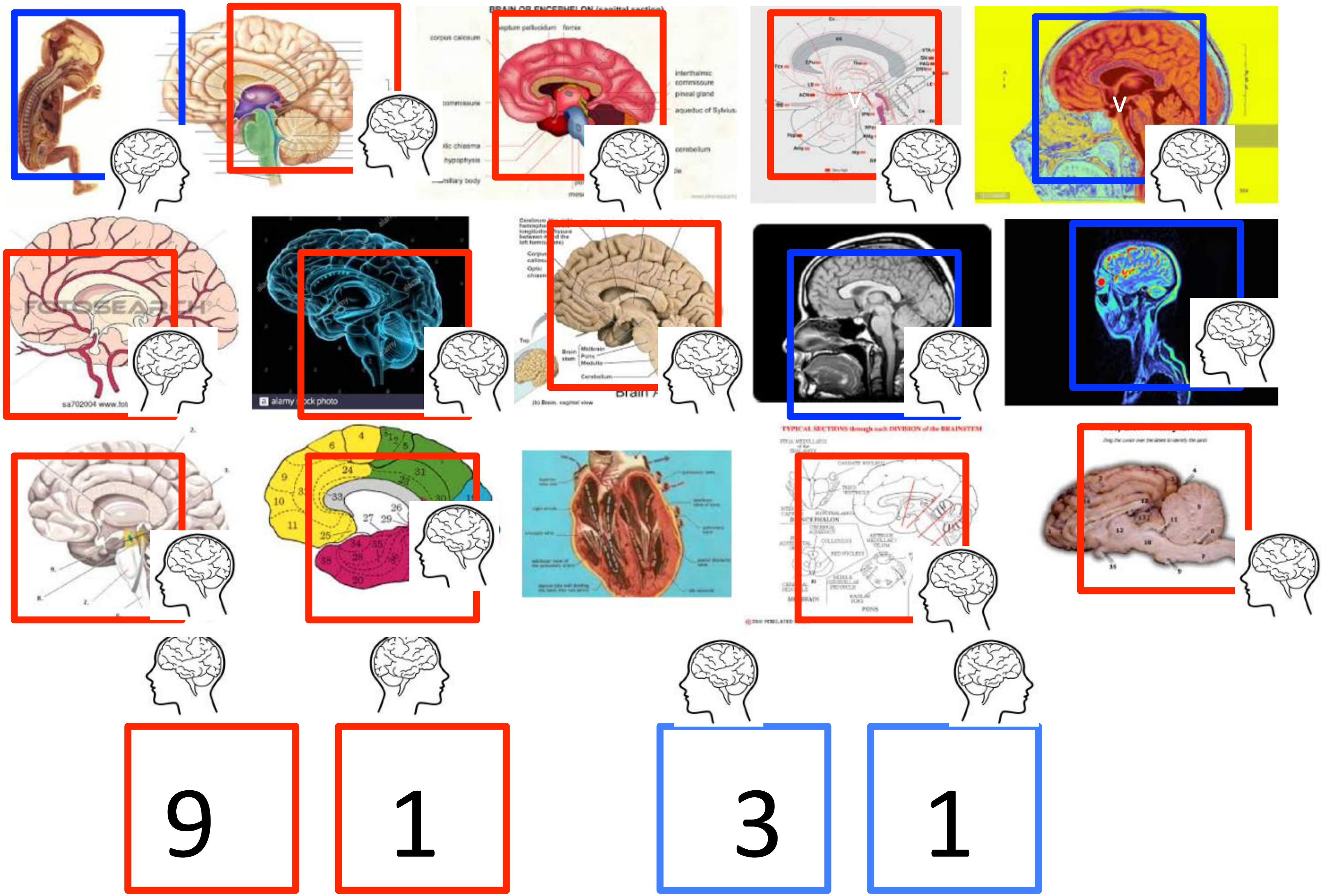
corpus callosum
fornix
interthalamic commissure
pineal gland
aqueduct of Sylvius
hypophysis
cerebellum
FOTOSEARCH
TYPICAL SECTIONS through each DIVISION of the BRAINSTEM
9
1
3
1

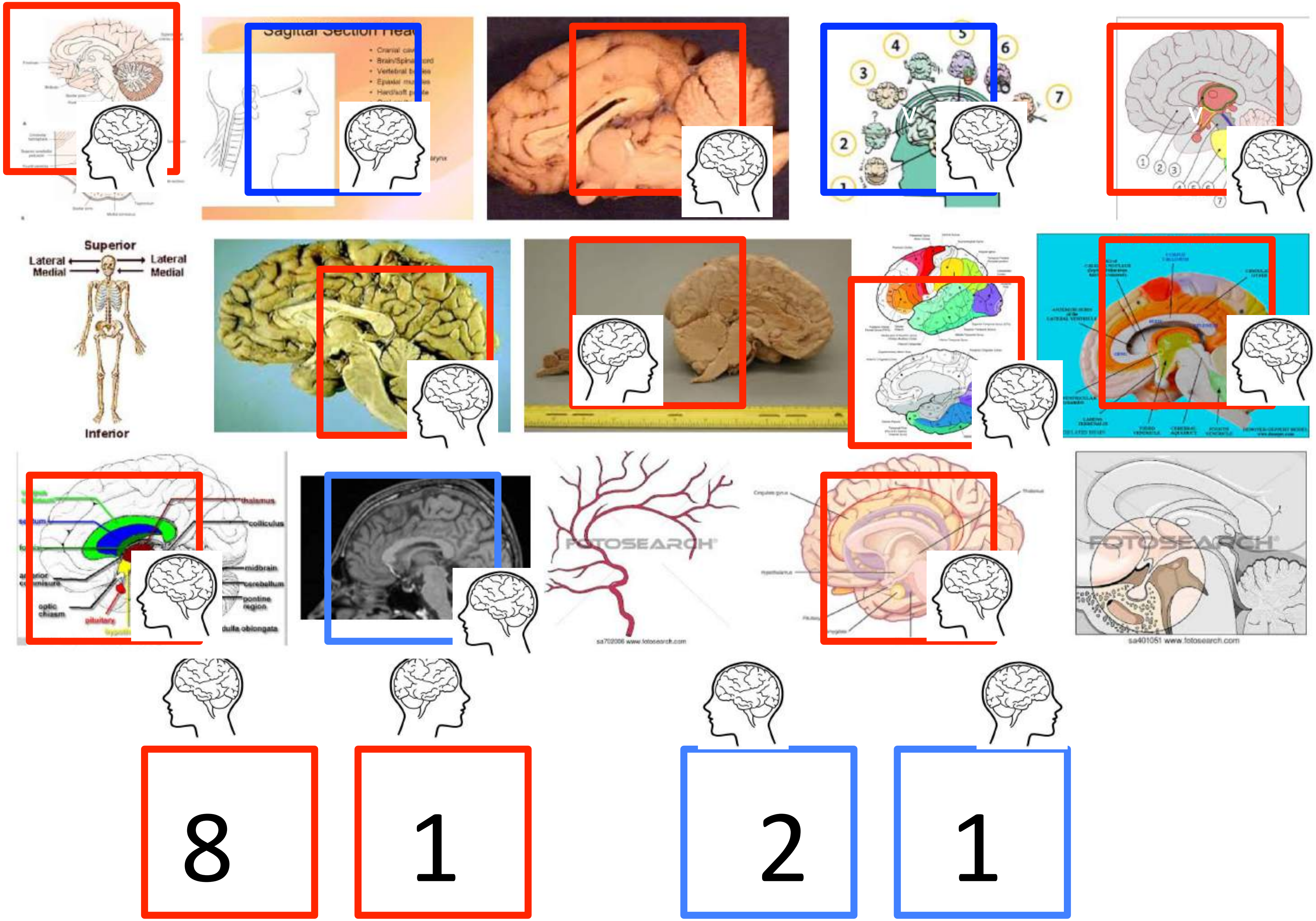

Superior
Lateral
Medial
Lateral
Medial
Inferior
8
1
2
1

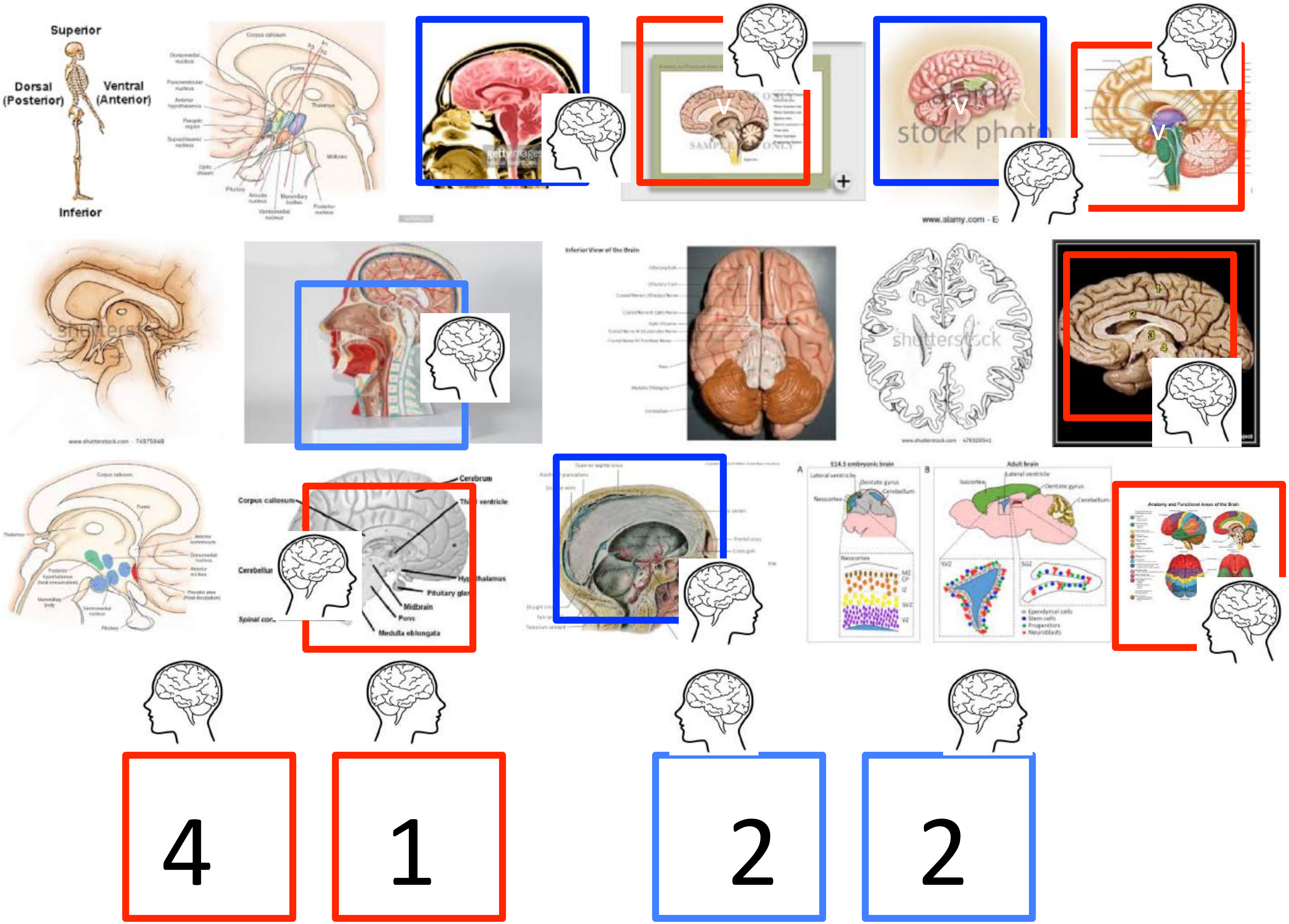

Superior
Dorsal (Posterior)
Ventral (Anterior)
Inferior
stock photo
www.alamy.com
Corpus callosum
Cerebrum
Cerebellum
Pituitary
Midbrain
Pons
Medulla oblongata
E14.5 embryonic brain
Adult brain
Anatomy and Functional Areas of the Brain
4
1
2
2

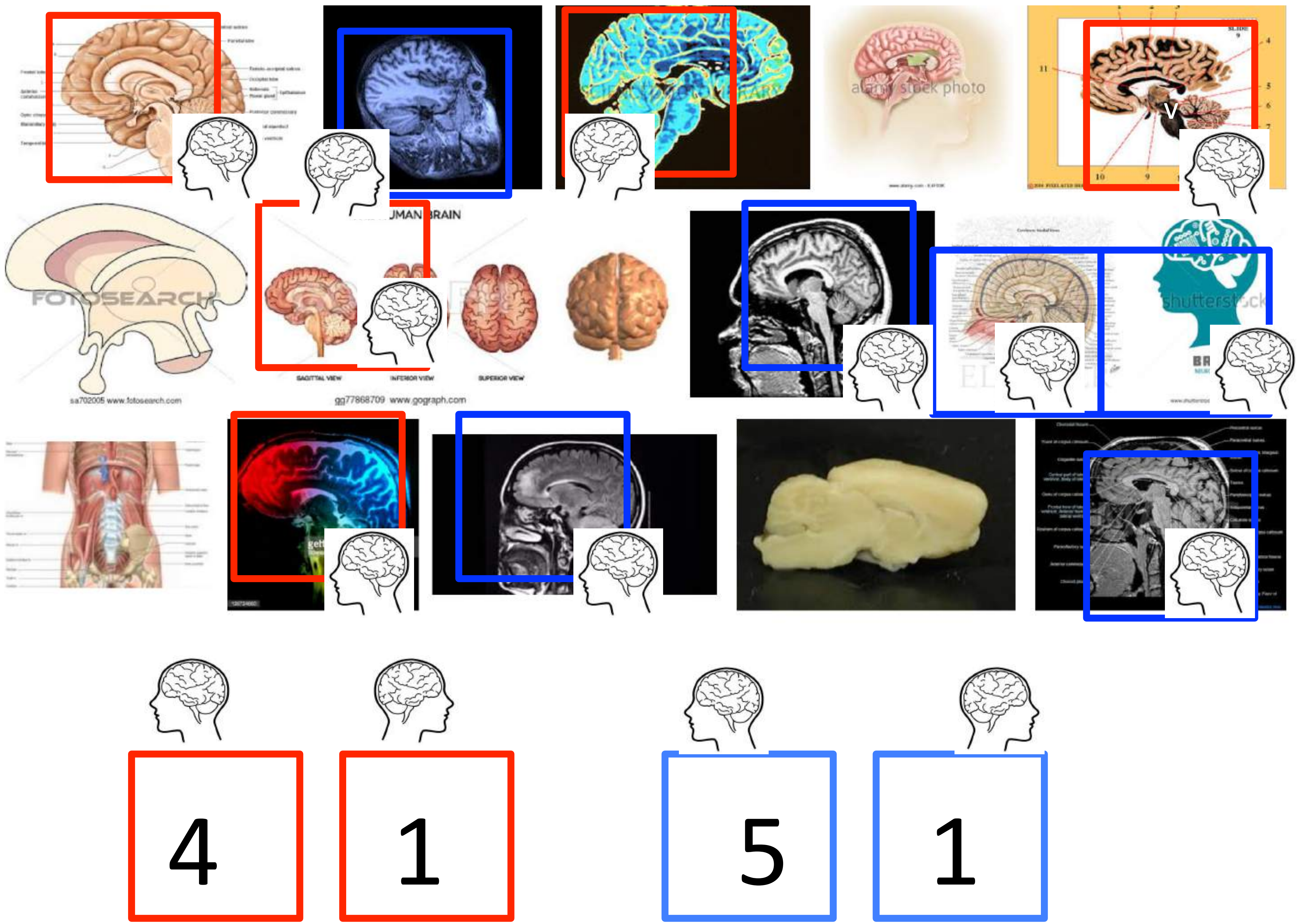

sa702005 www.fotosearch.com
SAGITTAL VIEW
INFERIOR VIEW
SUPERIOR VIEW
gg77868709 www.gograph.com
4
1
5
1

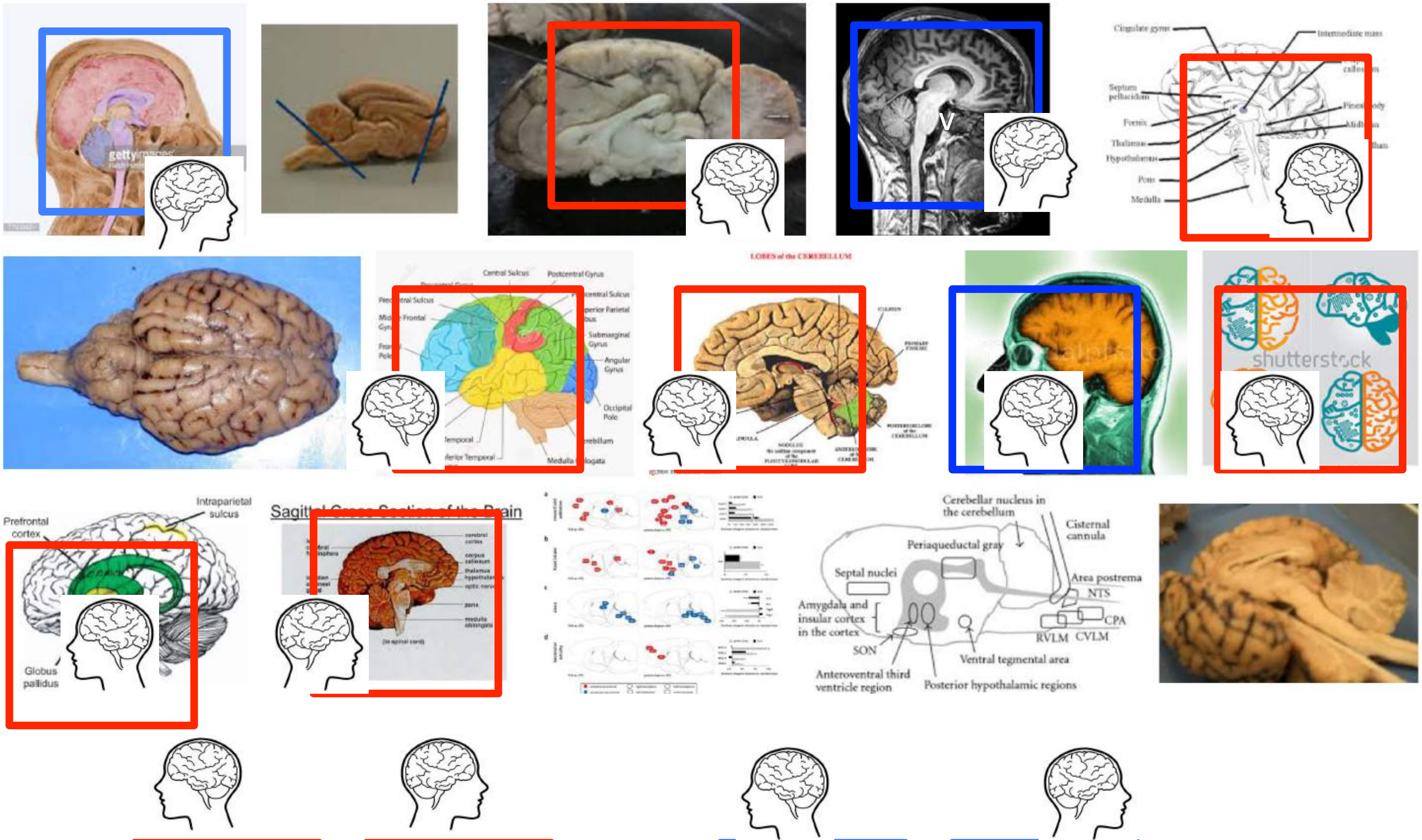
Prefrontal cortex
Intraparietal sulcus
Globus pallidus
Sagittal Cross Section of the Brain
shutterstock
Cerebellar nucleus in the cerebellum
Cisternal cannula
Periaqueductal gray
Septal nuclei
Area postrema
NTS
Amygdala and insular cortex in the cortex
CPA
RVLM CVLM
SON
Ventral tegmental area
Anteroventral third ventricle region
Posterior hypothalamic regions

6

1

1

2

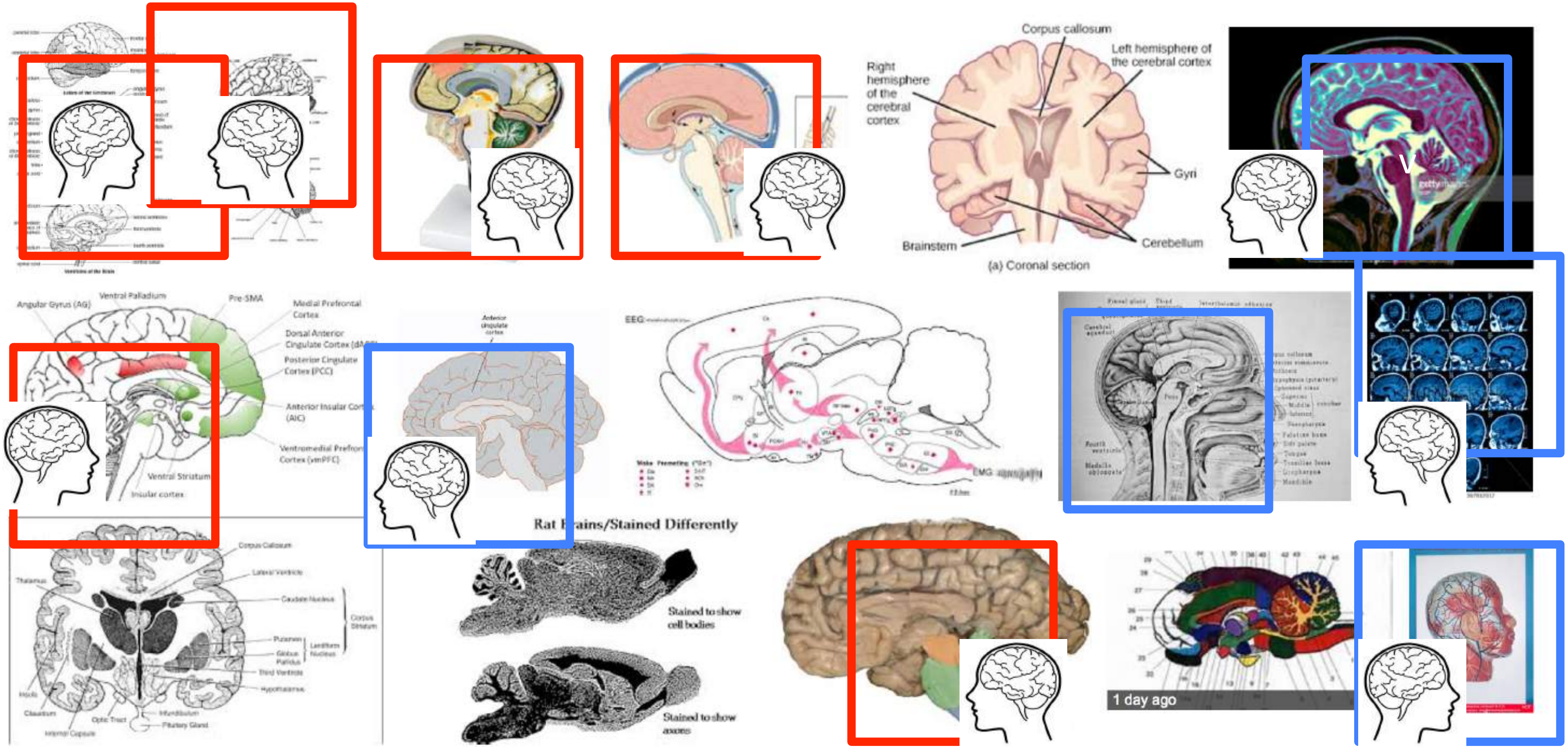

Corpus callosum
Left hemisphere of the cerebral cortex
Right hemisphere of the cerebral cortex
Gyri
Brainstem
Cerebellum
(a) Coronal section
Ventral Palladium
Angular Gyrus (AG)
Pre-SMA
Medial Prefrontal Cortex
Posterior Cingulate Cortex (PCC)
Anterior Insular Cortex (AIC)
Ventral Striatum
Insular cortex
EEG
EMG
Corpus Callosum
Lateral Ventricle
Caudate Nucleus
Thalamus
Putamen
Third Ventricle
Hypothalamus
Optic Tract
Pituitary Gland
Internal Capsule
Claustrum
Stained to show cell bodies
Stained to show axons
1 day ago

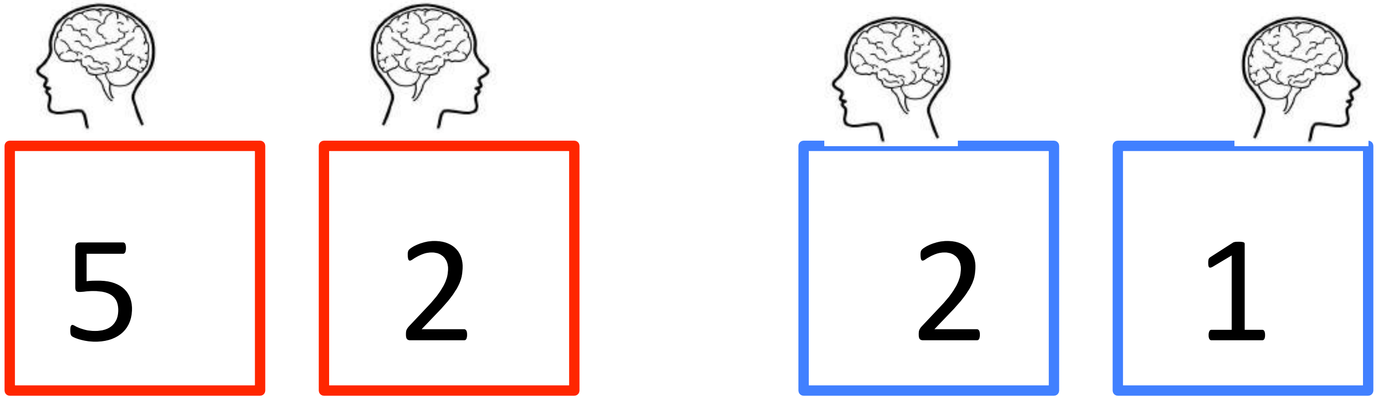

5
2
2
1

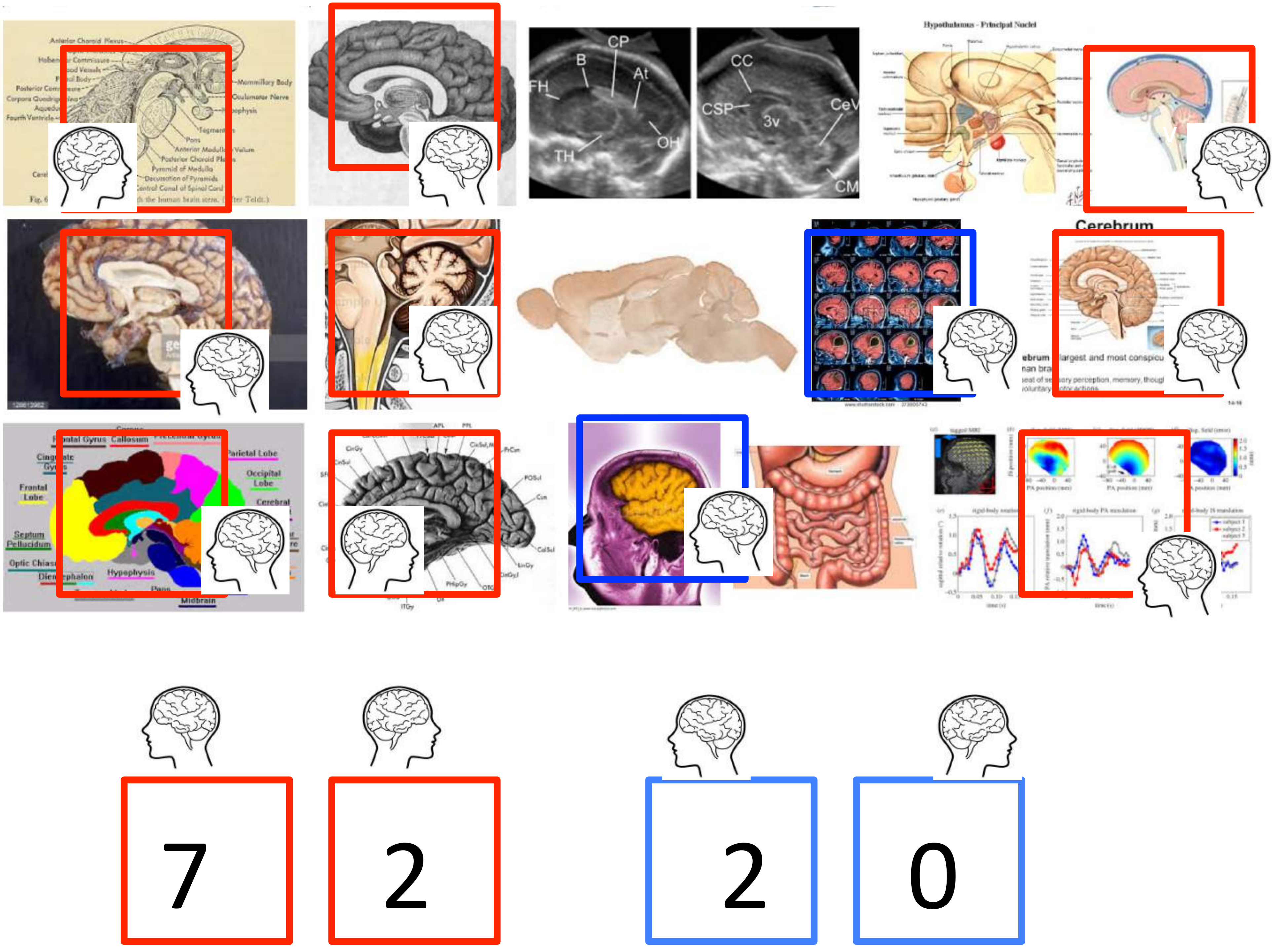

Hypothalamus - Principal Nuclei
CP
B
FH
At
OH
TH
CC
CSP
3v
CeV
CM
Cerebrum
Frontal Lobe
Occipital Lobe
Parietal Lobe
Septum Pellucidum
Optic Chiasm
Hypophysis
Midbrain
7
2
2
0

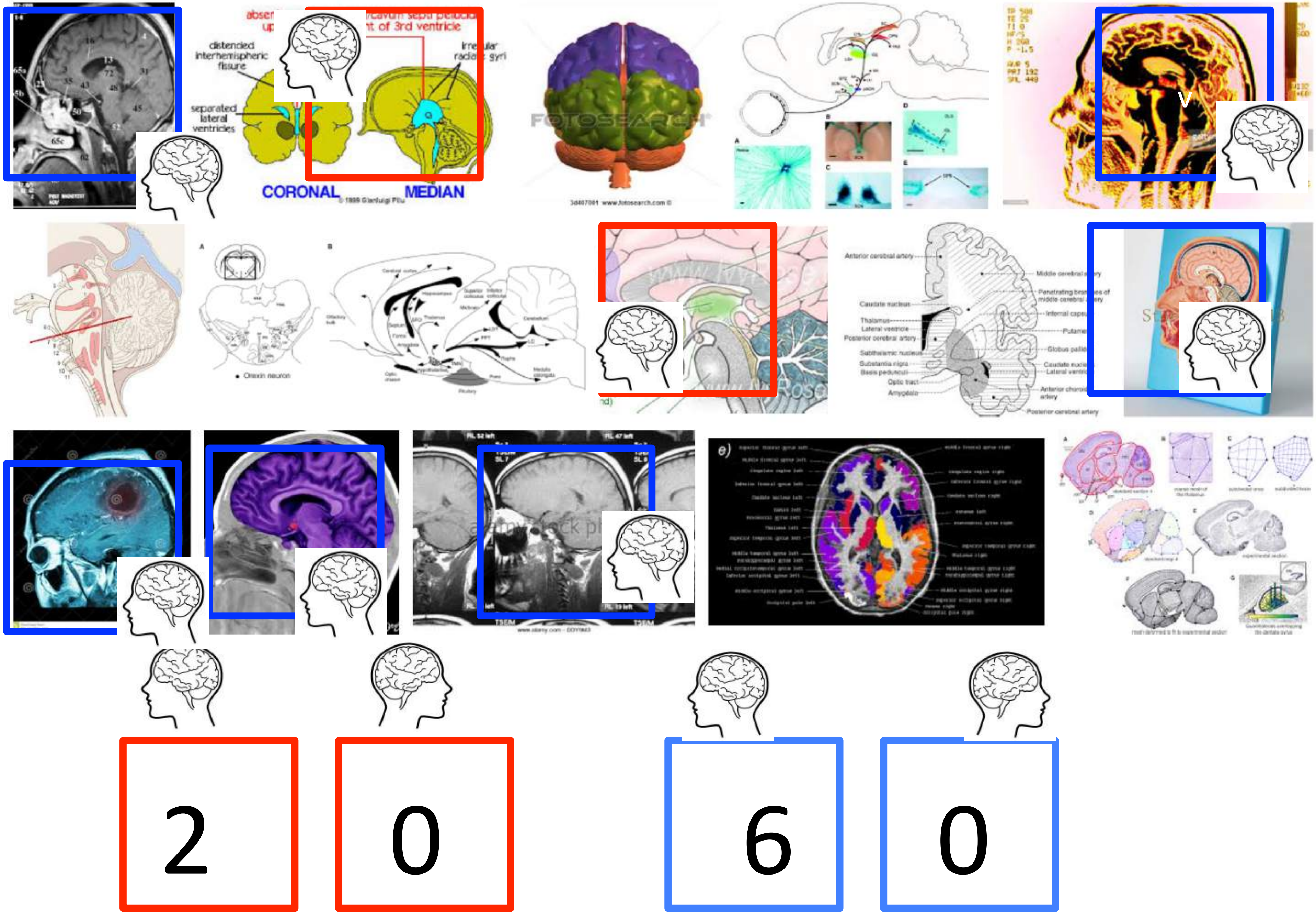
distended interhemispheric fissure
separated lateral ventricles
CORONAL
MEDIAN
2
0
6
0

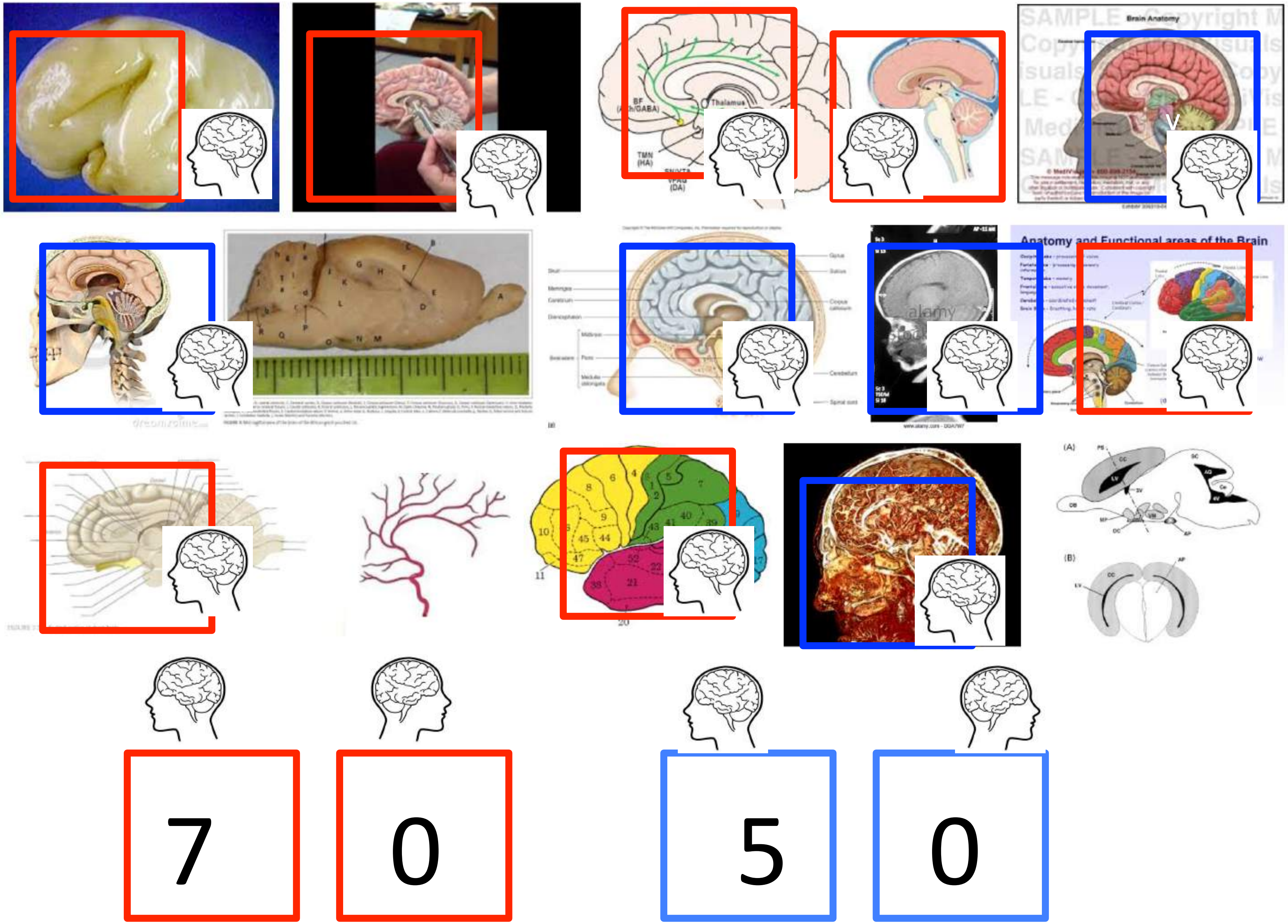

Brain Anatomy
Anatomy and Functional areas of the Brain
Thalamus
7
0
5
0

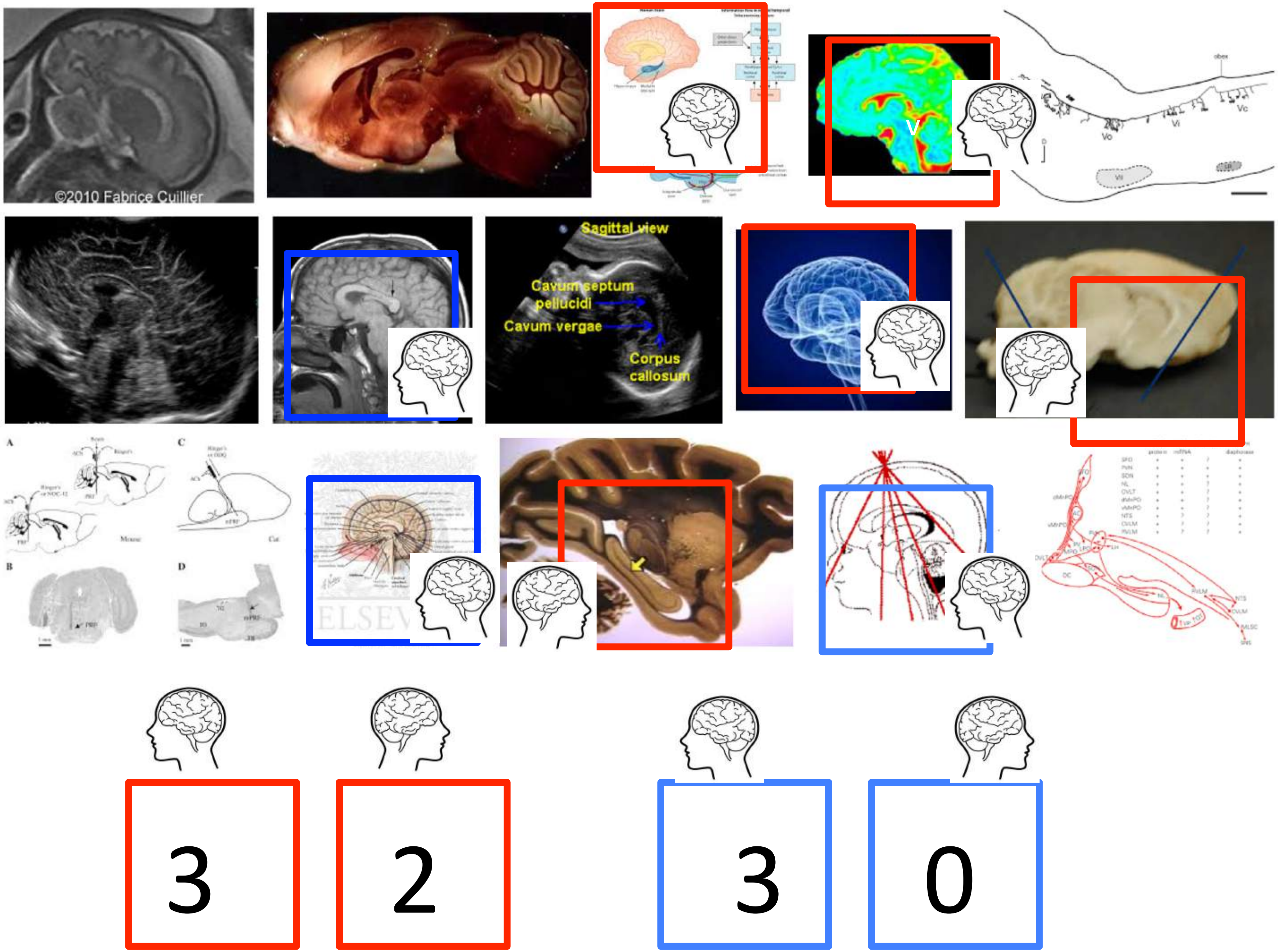
©2010 Fabrice Cuillier
V
obex
Vo
Vi
Vc
VII
Sagittal view
Cavum septum pellucidi
Cavum vergae
Corpus callosum
Mouse
Cat
ELSEV
3
2
3
0

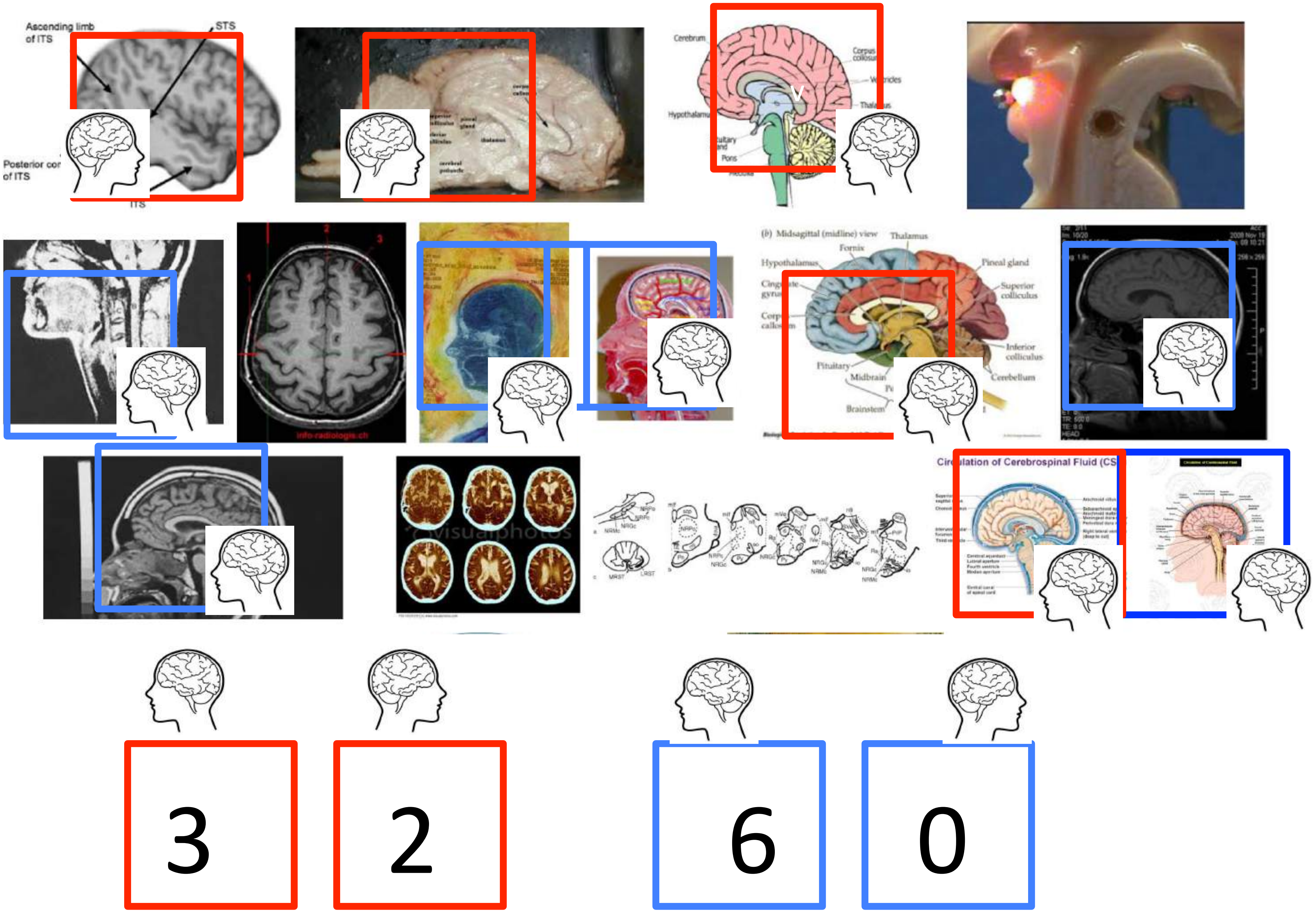

Ascending limb of ITS
STS
Posterior cor of ITS
ITS
Cerebrum
Hypothalamus
Pons
(b) Midsagittal (midline) view
Thalamus
Fornix
Hypothalamus
Pineal gland
Superior colliculus
Inferior colliculus
Cerebellum
Pituitary
Midbrain
Brainstem
info-radiologie.ch
3
2
6
0

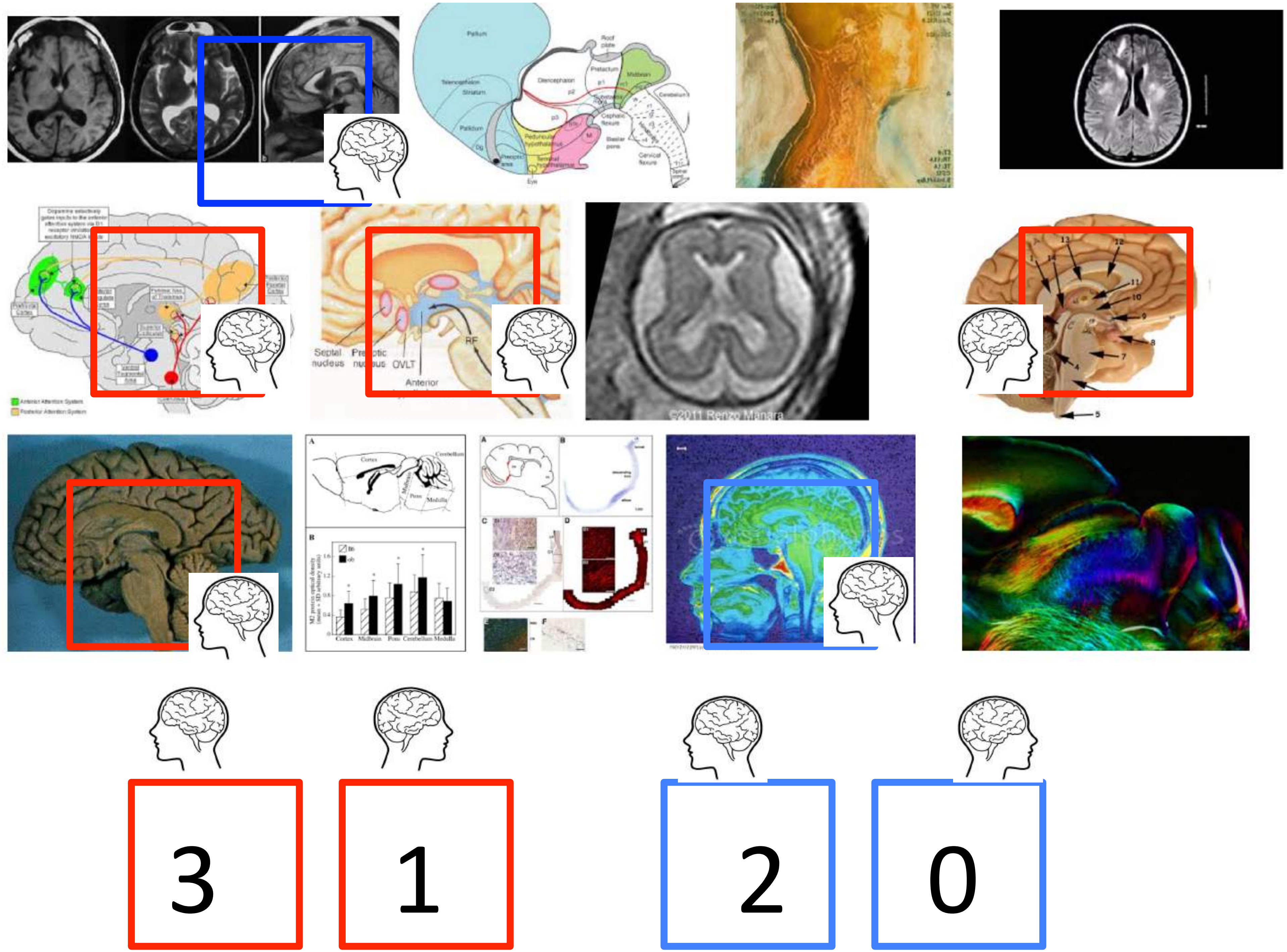
3
1
2
0

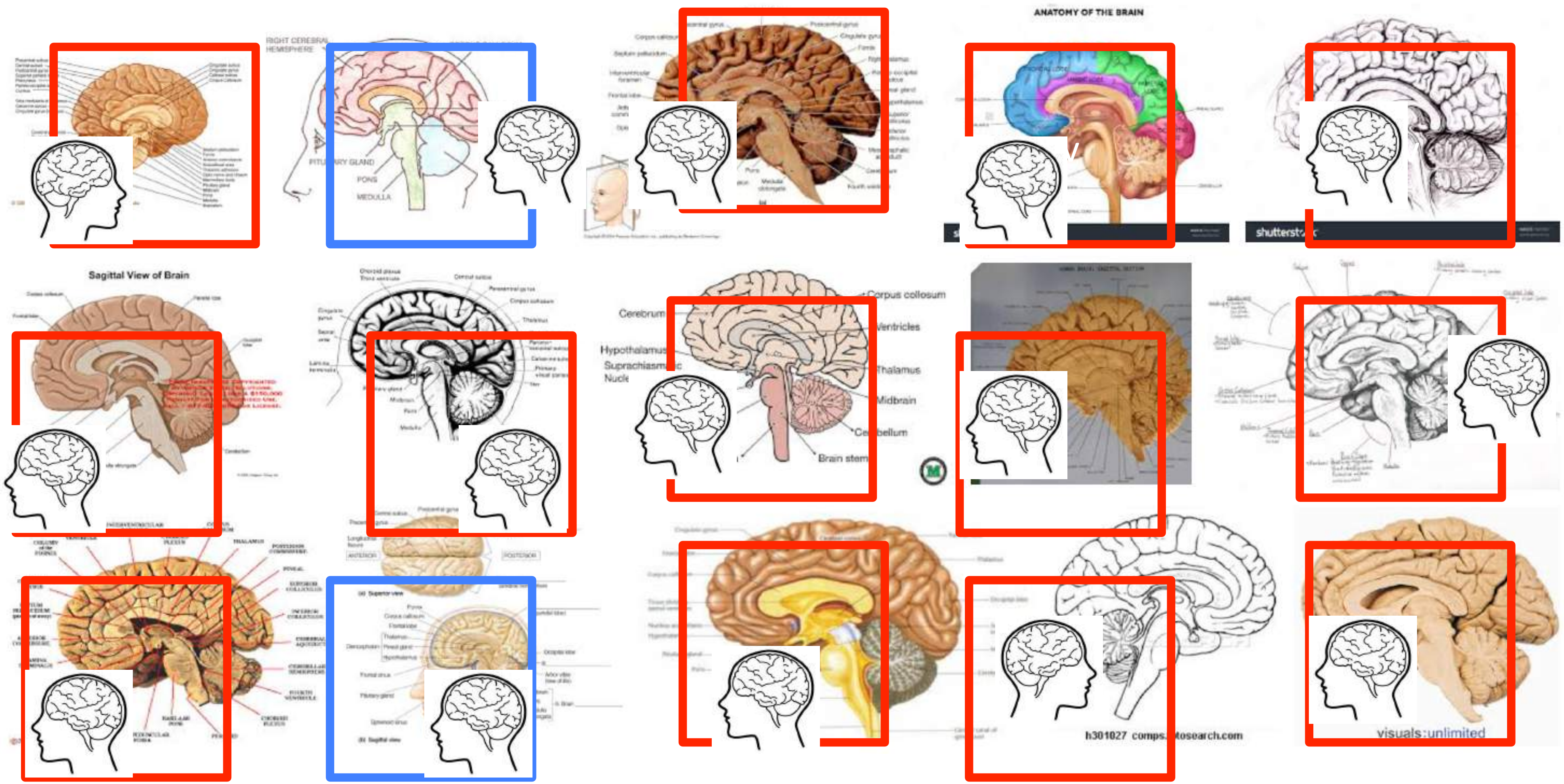
ANATOMY OF THE BRAIN
Sagittal View of Brain
Cerebrum
Corpus collosum
Ventricles
Thalamus
Midbrain
Brain stem
visuals:unlimited

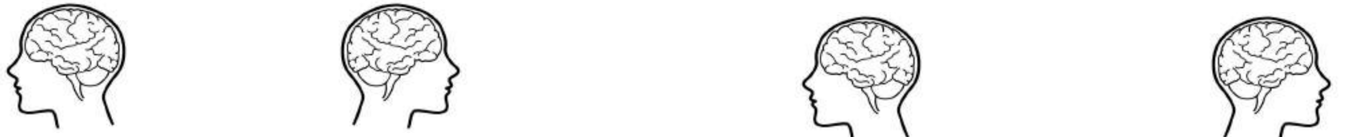

11

2

2

0

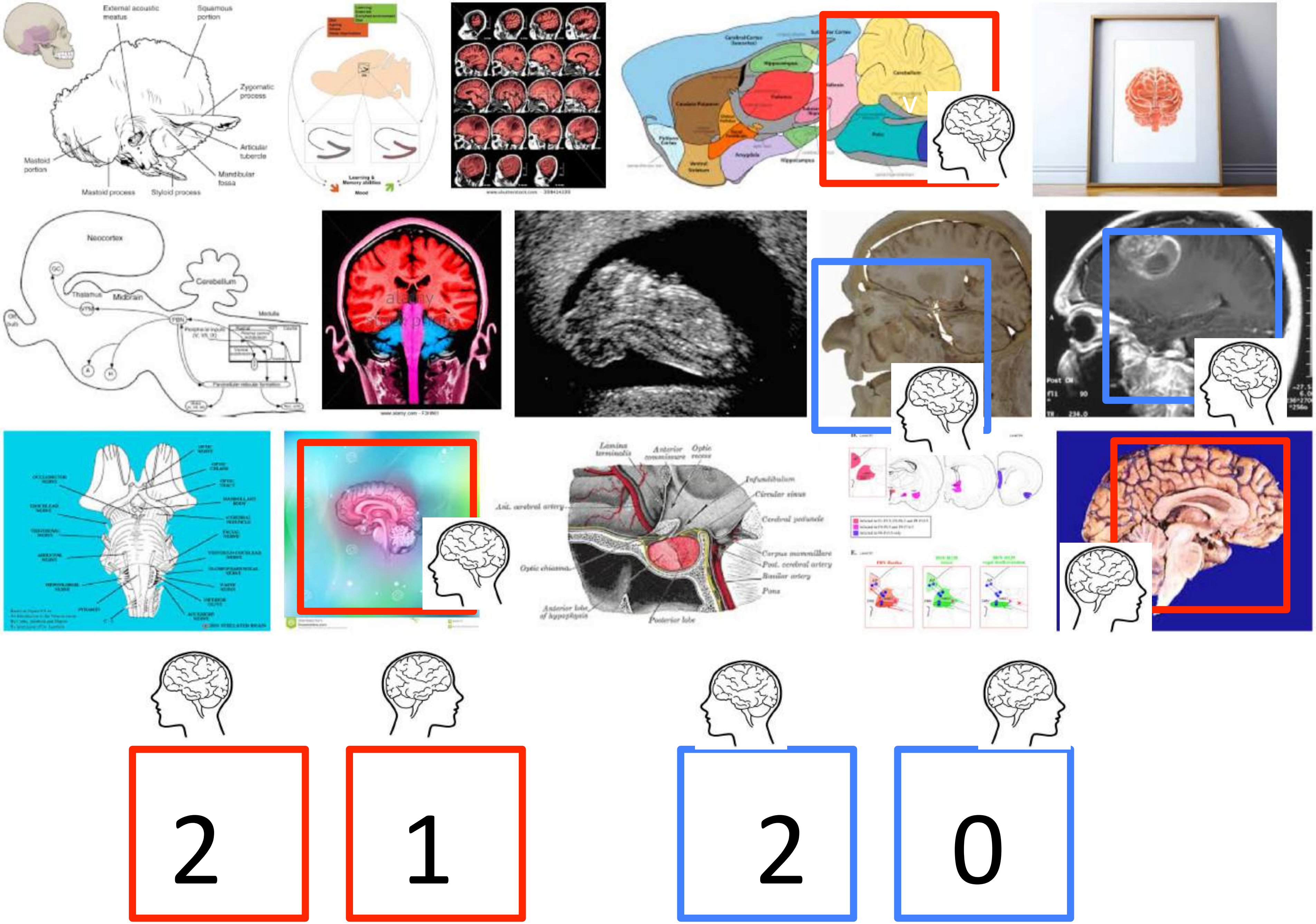
External acoustic meatus
Squamous portion
Zygomatic process
Articular tubercle
Mandibular fossa
Mastoid portion
Mastoid process
Styloid process
Neocortex
Cerebellum
Thalamus
Midbrain
Medulla
Lamina terminalis
Anterior commissure
Optic recess
Infundibulum
Circular sinus
Cerebral peduncle
Ant. cerebral artery
Optic chiasma
Corpus mammillare
Post. cerebral artery
Basilar artery
Pons
Anterior lobe of hypophysis
Posterior lobe
2
1
2
0

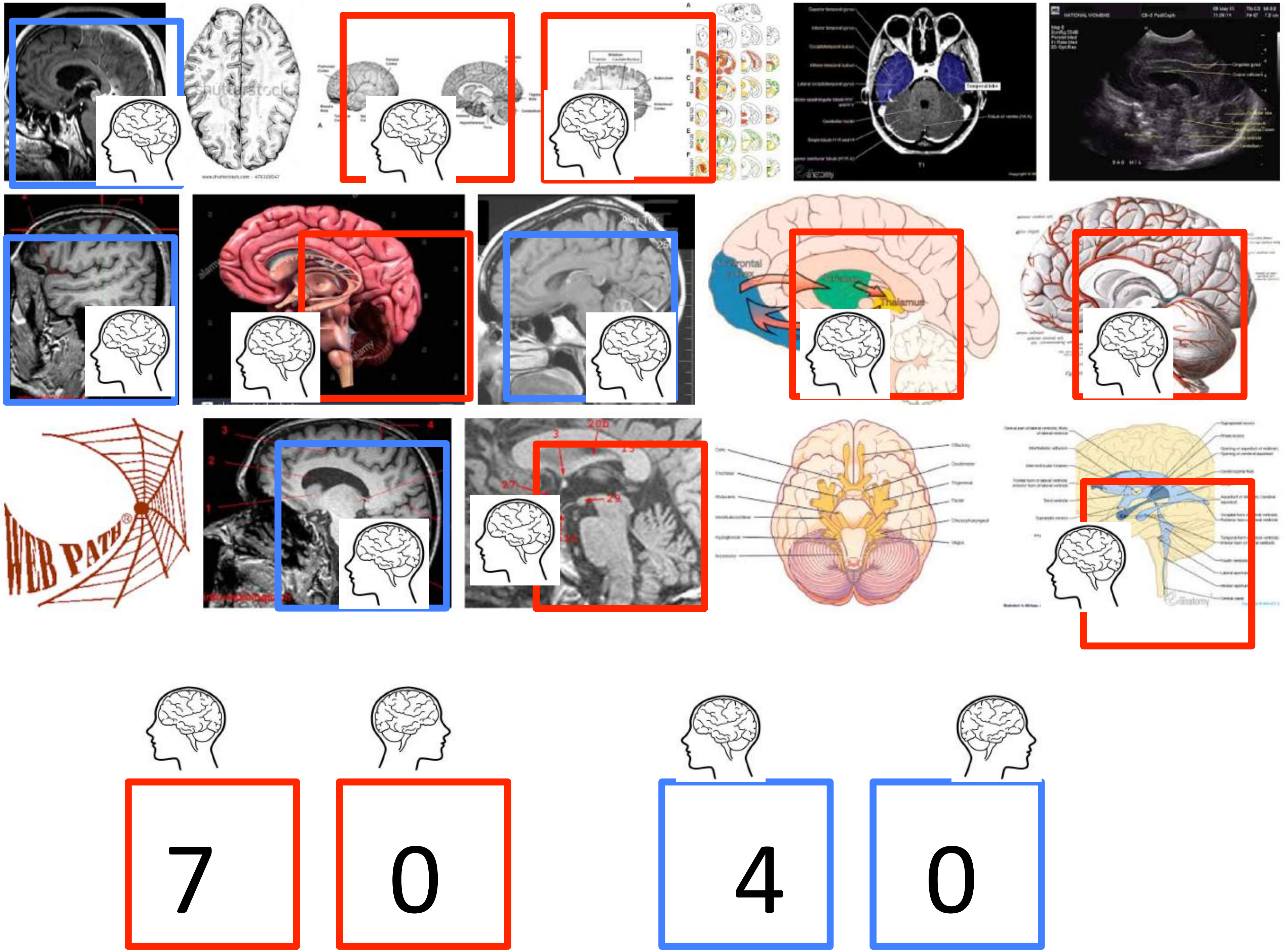

WEB PATH
7
0
4
0

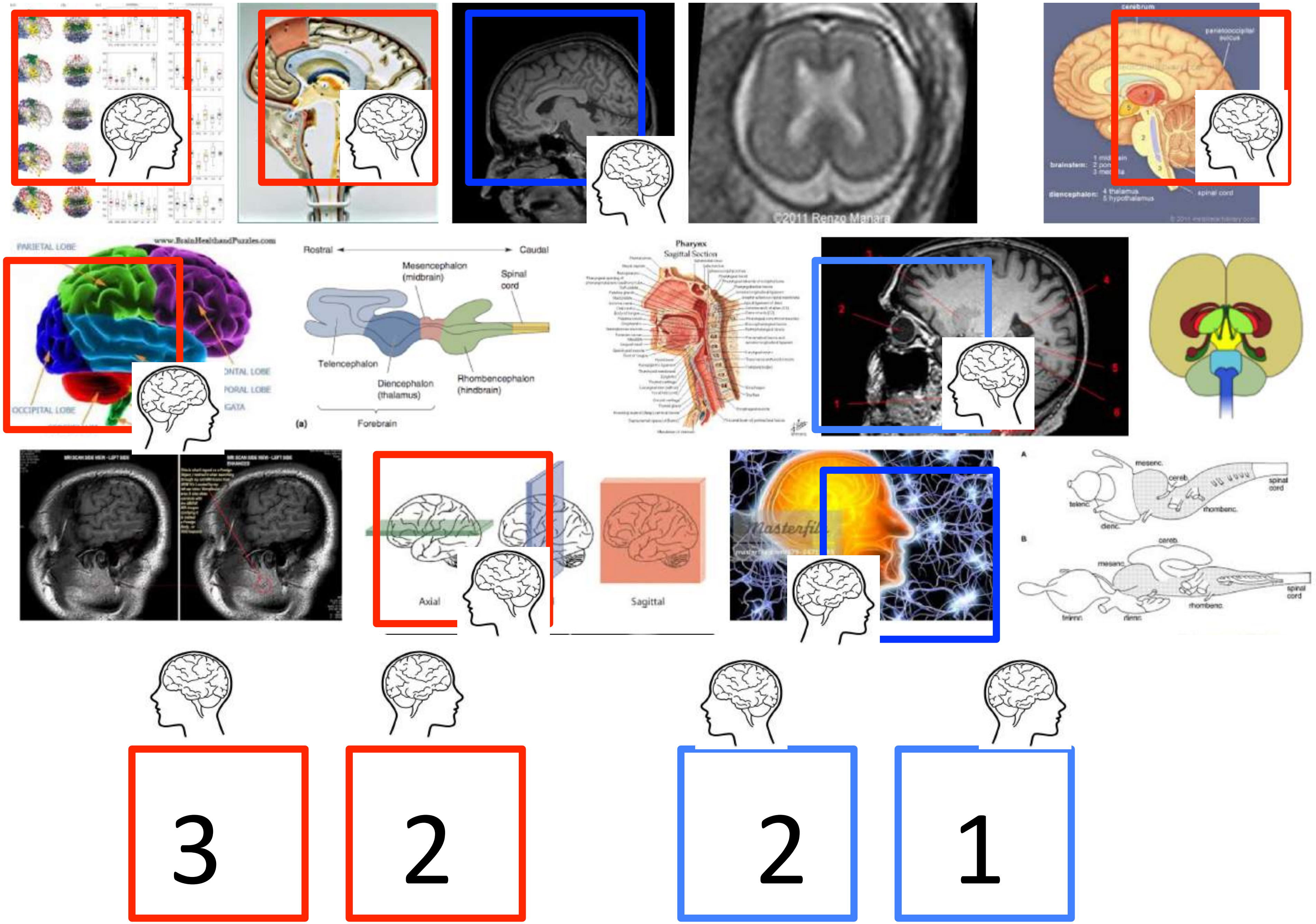

cerebrum
brainstem:
diencephalon:
4 thalamus
5 hypothalamus
spinal cord
©2011 Renzo Manara
PARIETAL LOBE
www.BrainHealthandPuzzles.com
OCCIPITAL LOBE
Rostral
Caudal
Mesencephalon (midbrain)
Spinal cord
Telencephalon
Diencephalon (thalamus)
Rhombencephalon (hindbrain)
(a)
Forebrain
Pharynx
Sagittal Section
Axial
Sagittal
A
mesenc.
cereb.
telenc.
dienc.
rhombenc.
spinal cord
B
3
2
2
1

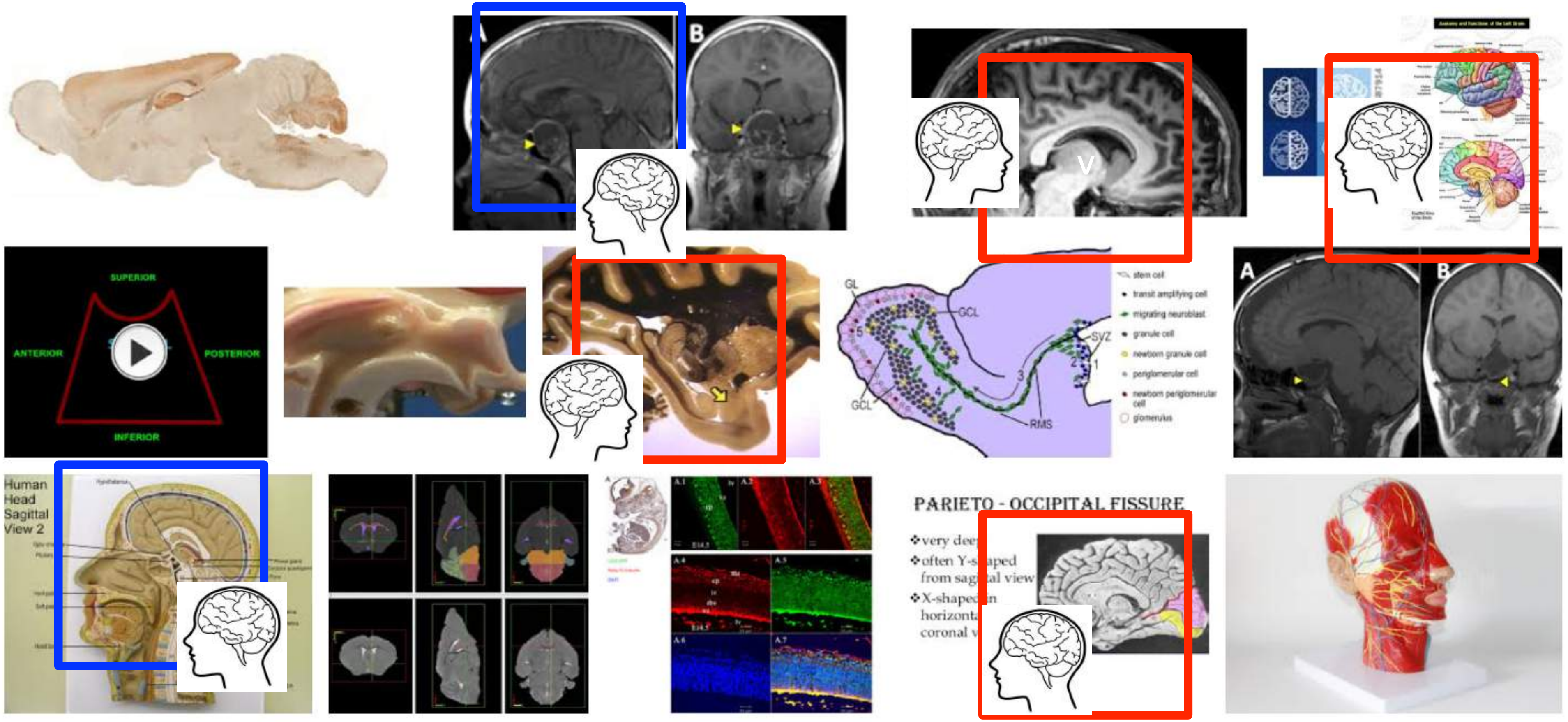

SUPERIOR
ANTERIOR
POSTERIOR
INFERIOR
Human Head Sagittal View 2
GL
GCL
SVZ
RMS
stem cell
transit amplifying cell
migrating neuroblast
granule cell
newborn granule cell
periglomerular cell
newborn periglomerular cell
glomerulus
PARIETO - OCCIPITAL FISSURE

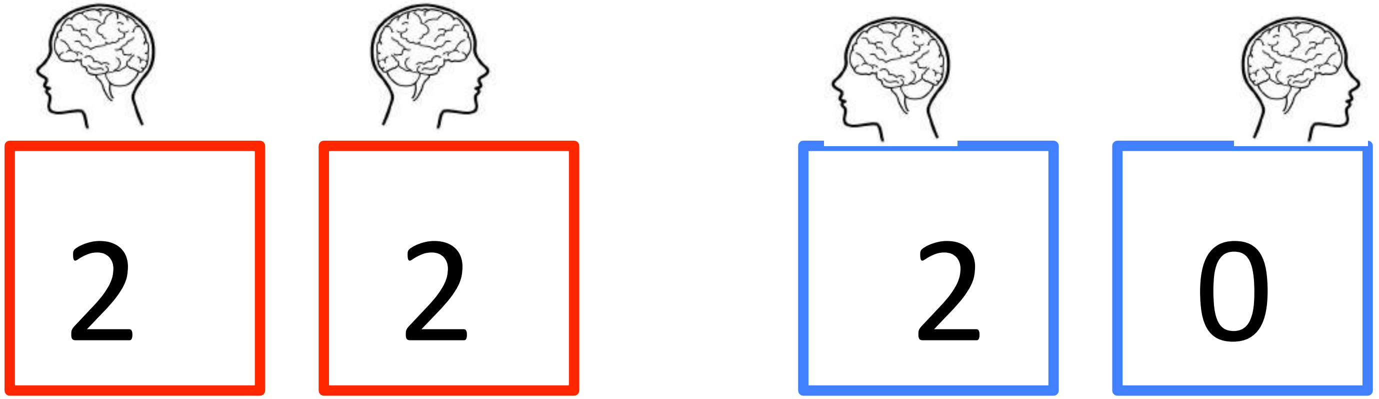

2
2
2
0

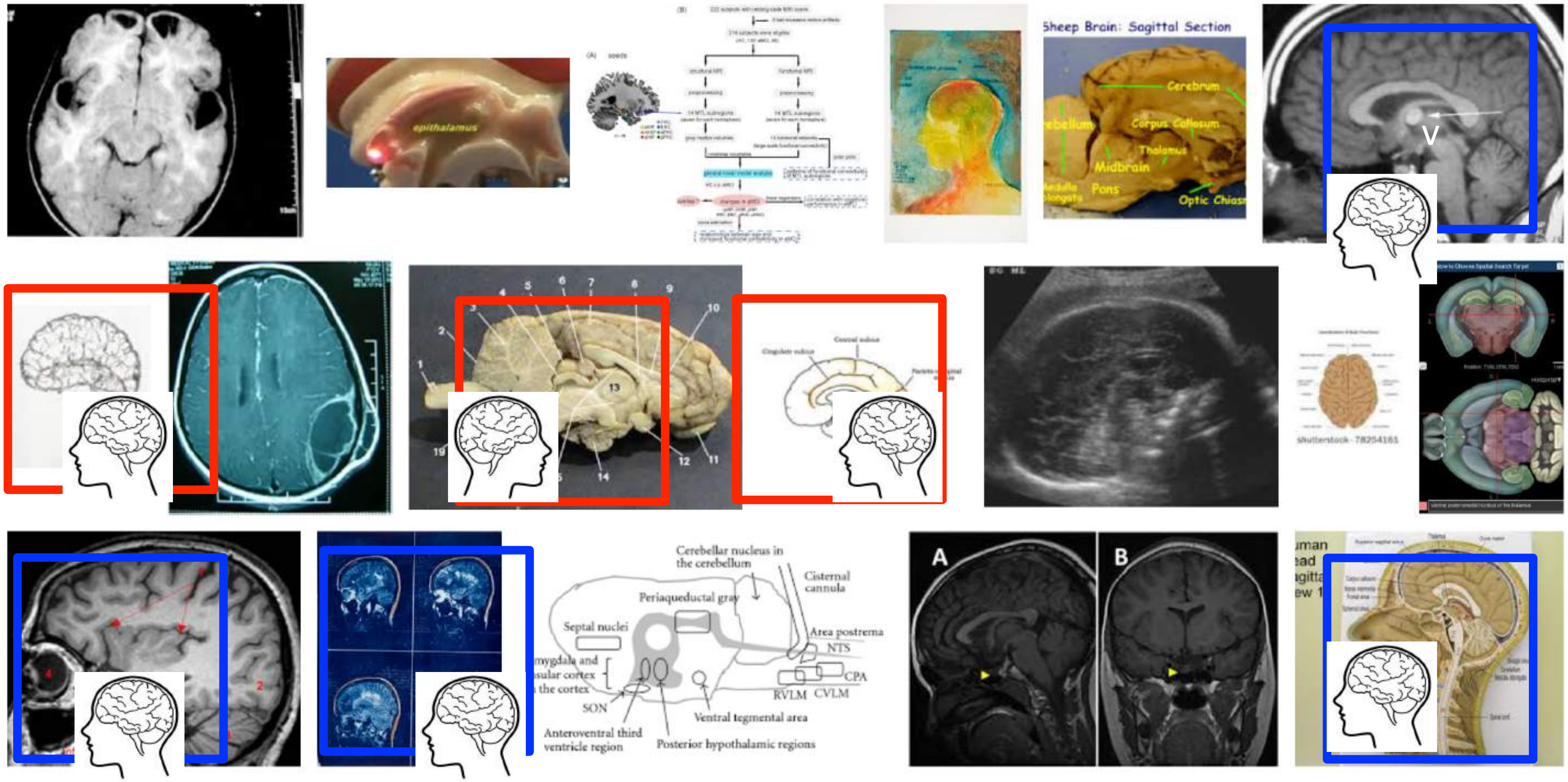
epithalamus
Sheep Brain: Sagittal Section
Cerebrum
Corpus Callosum
Thalamus
Midbrain
Pons
V
Cingulate sulcus
Central sulcus
Cerebellar nucleus in the cerebellum
Cisternal cannula
Periaqueductal gray
Septal nuclei
Area postrema
NTS
CPA
RVLM
CVLM
SON
Ventral tegmental area
Anteroventral third ventricle region
Posterior hypothalamic regions
A
B

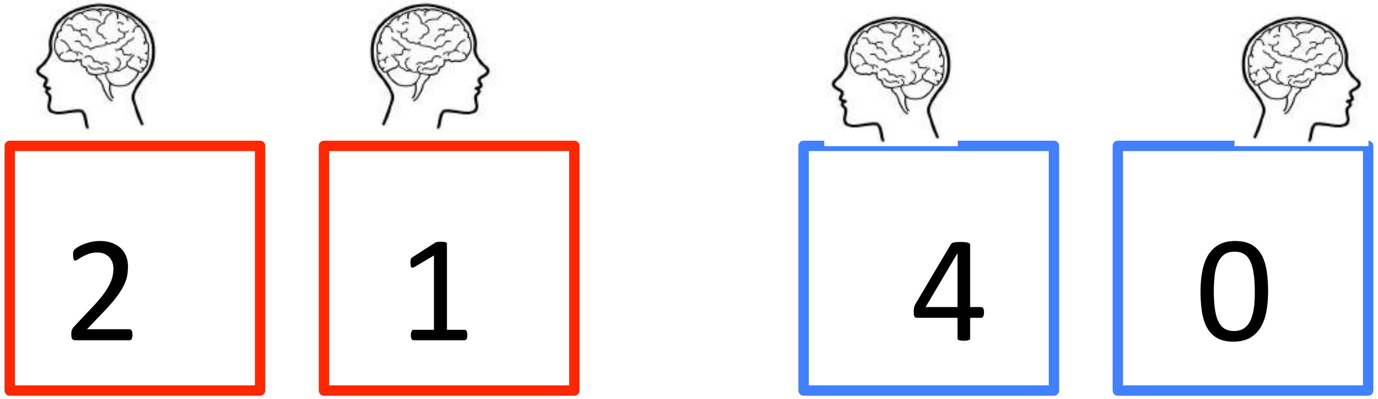
2
1
4
0

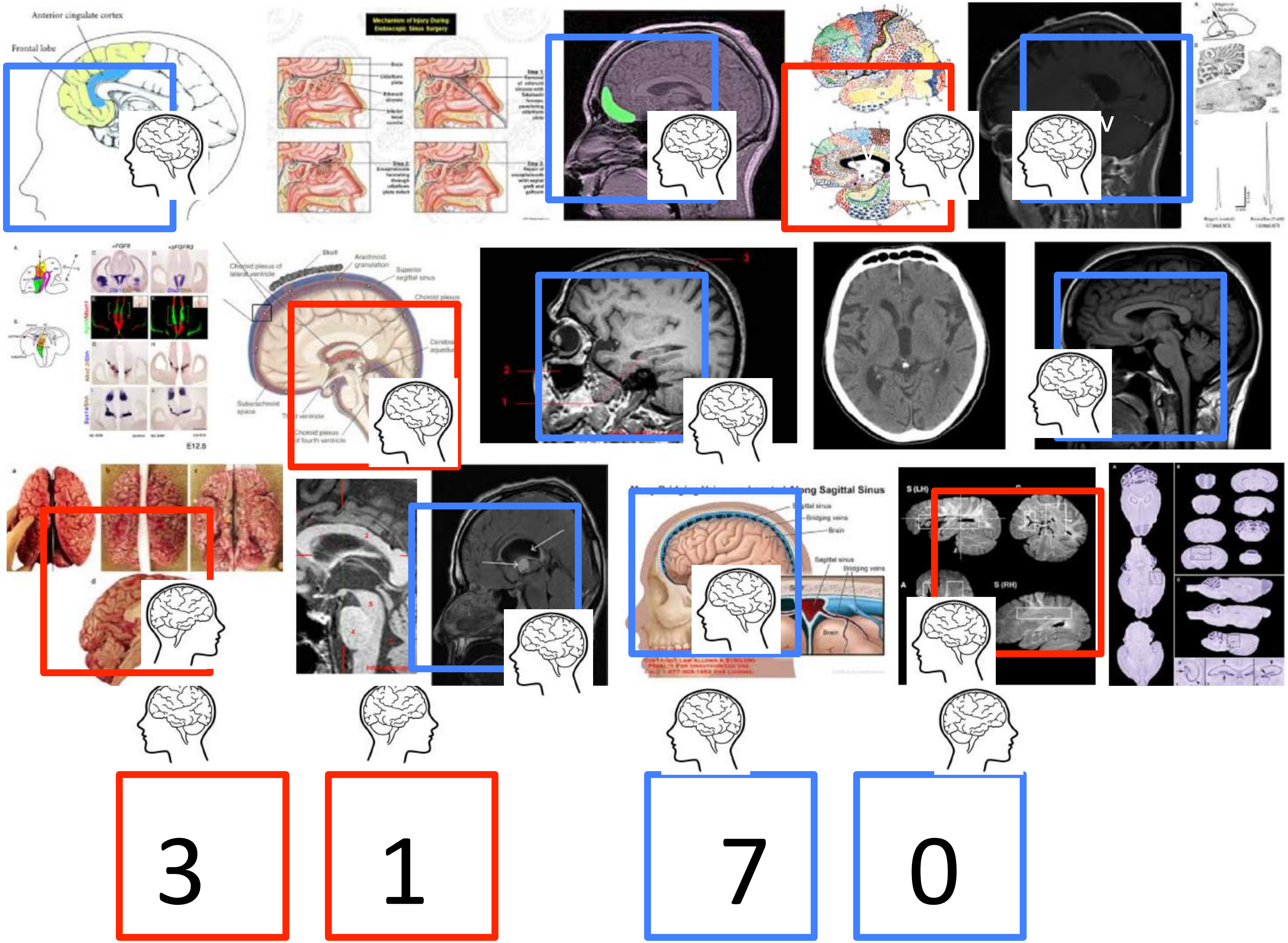
Anterior cingulate cortex
Frontal lobe
Mechanism of Injury During Endoscopic Sinus Surgery
Skull
Arachnoid granulation
Superior sagittal sinus
Choroid plexus of lateral ventricle
Choroid plexus
Subarachnoid space
E12.5
Sagittal Sinus
Bridging veins
Brain
S (LH)
S (RH)
3
1
7
0

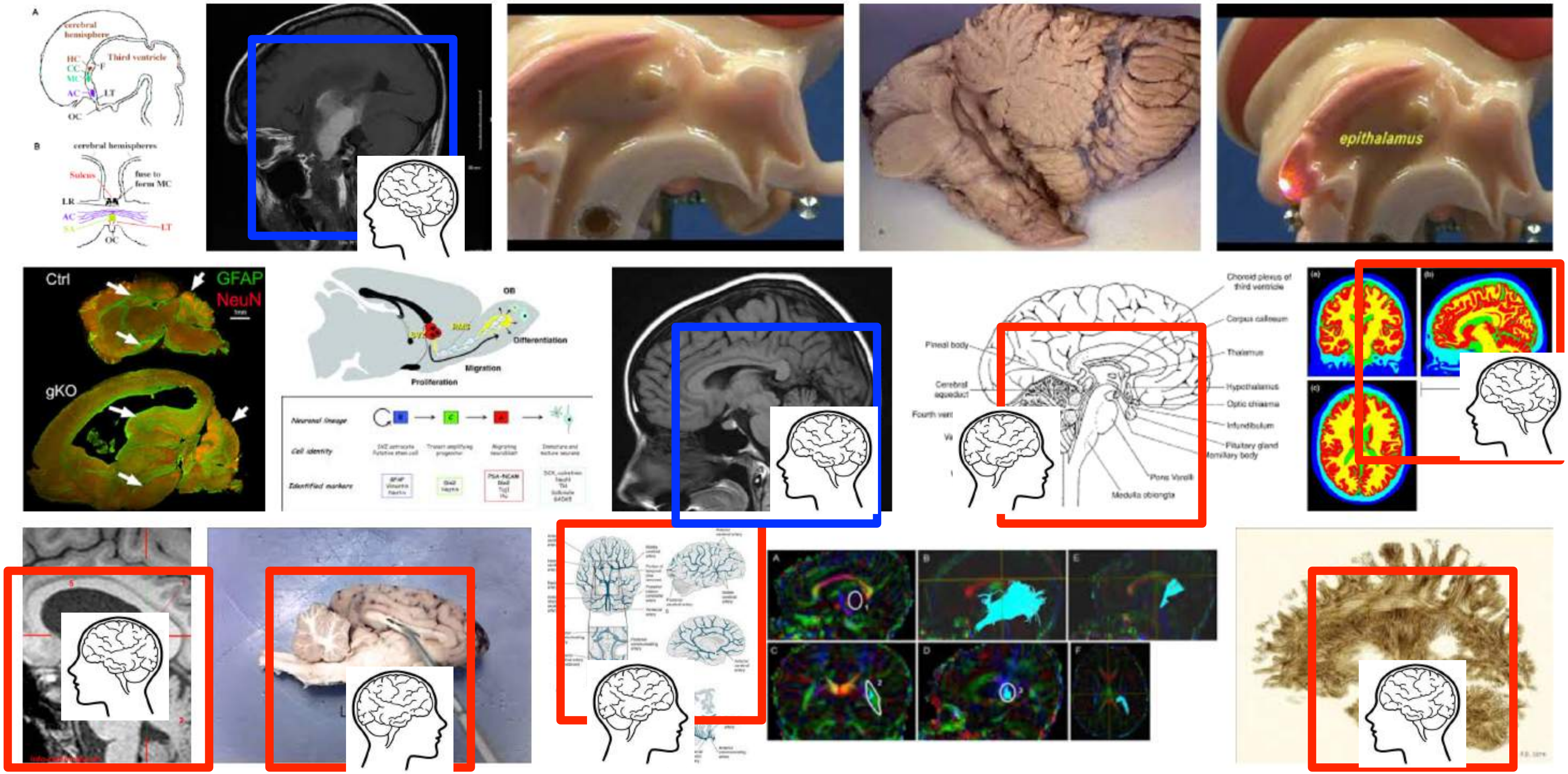
cerebral hemisphere
Third ventricle
cerebral hemispheres
Sulcus
fuse to form MC
epithalamus
Ctrl
GFAP
NeuN
gKO
OB
Differentiation
Migration
Proliferation
Pineal body
Choroid plexus of third ventricle
Corpus callosum
Thalamus
Hypothalamus
Optic chiasma
Infundibulum
Pituitary gland
Pons Varolii
Medulla oblongata

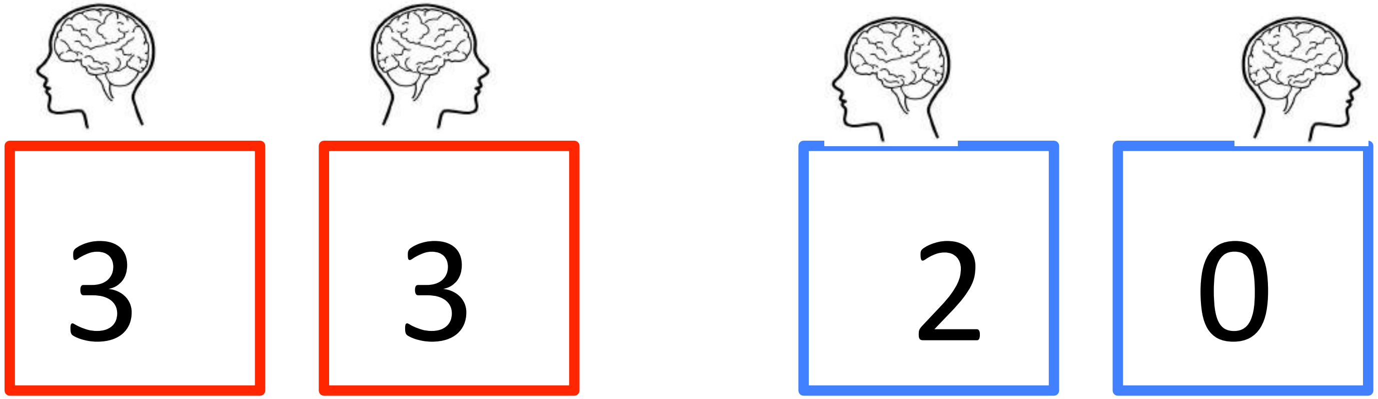
3
3
2
0

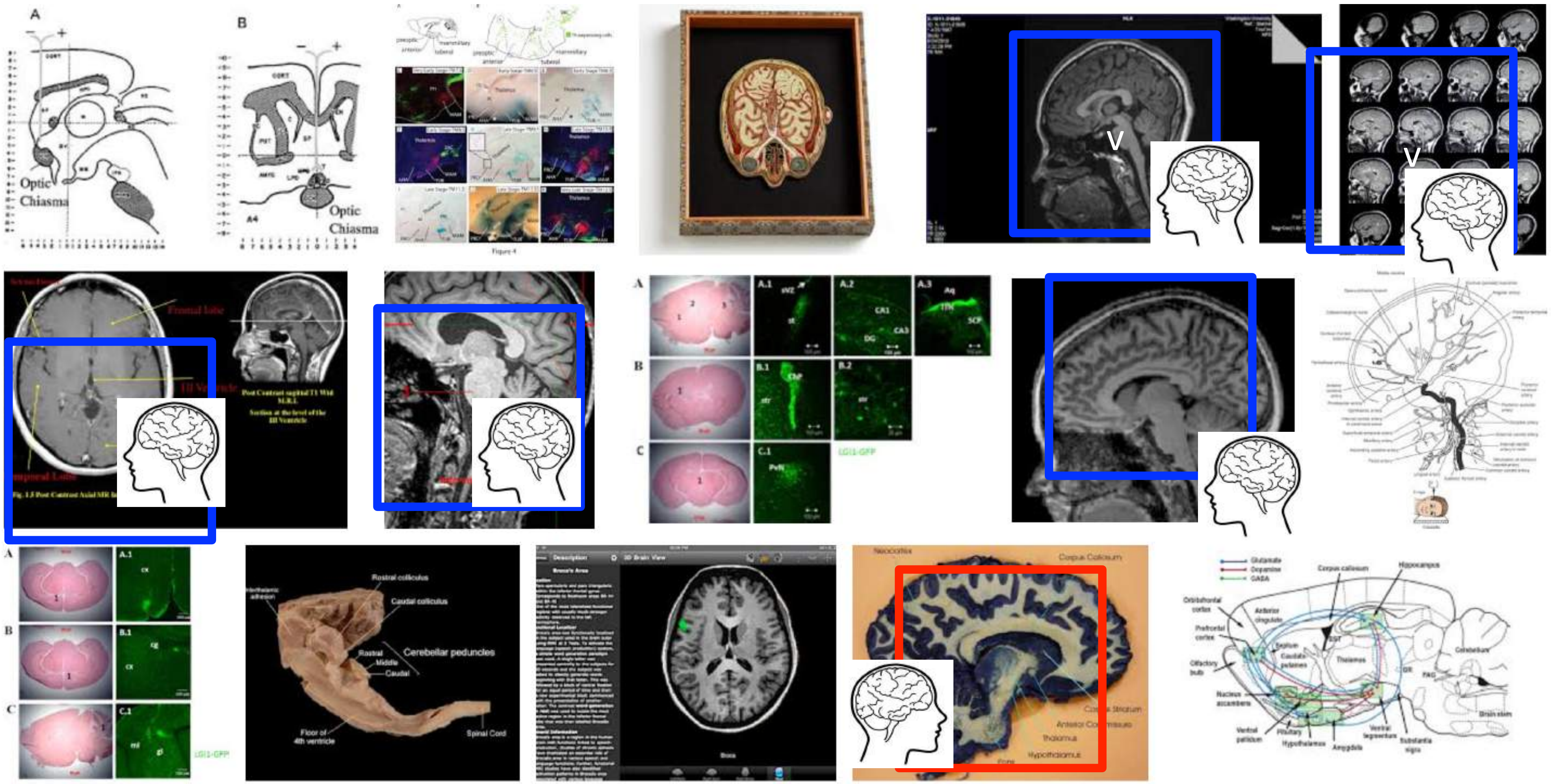
Optic Chiasma
Optic Chiasma
Rostral colliculus
Caudal colliculus
Cerebellar peduncles
Rostral
Middle
Caudal
Floor of 4th ventricle
Spinal Cord

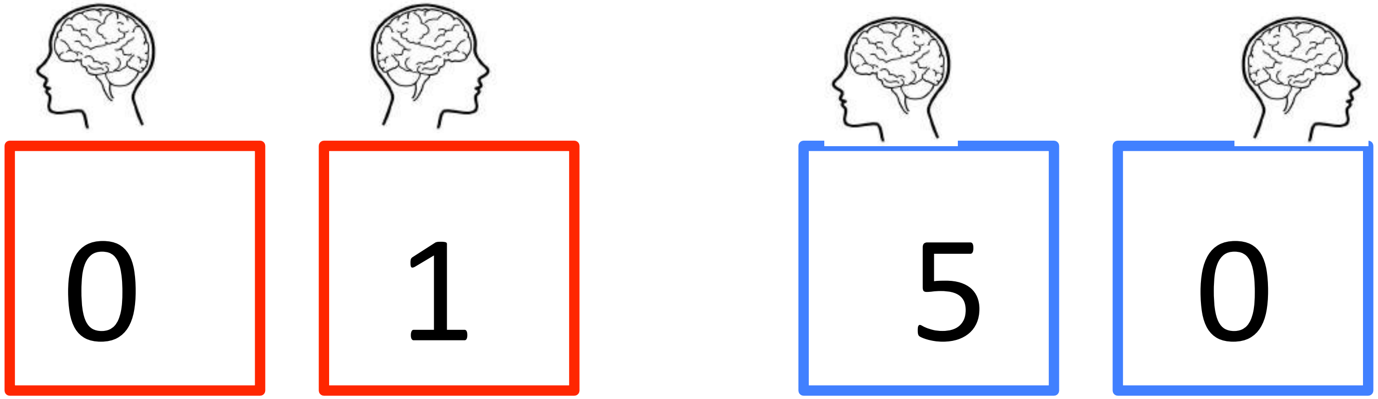
0
1
5
0

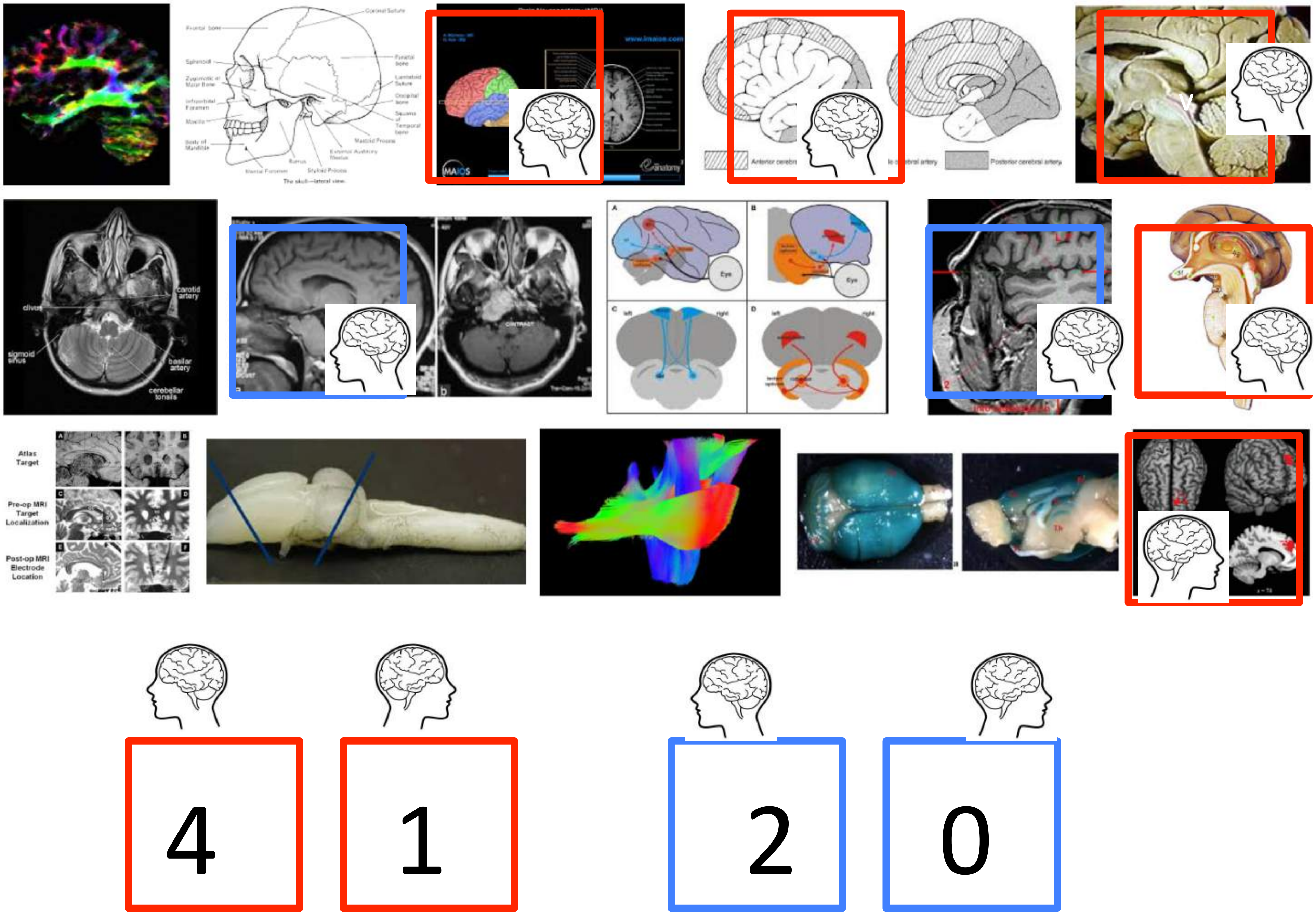

www.imaios.com
The skull—lateral view
carotid artery
clivus
sigmoid sinus
basilar artery
cerebellar tonsils
Atlas Target
Pre-op MRI Target Localization
Post-op MRI Electrode Location
Posterior cerebral artery
4
1
2
0

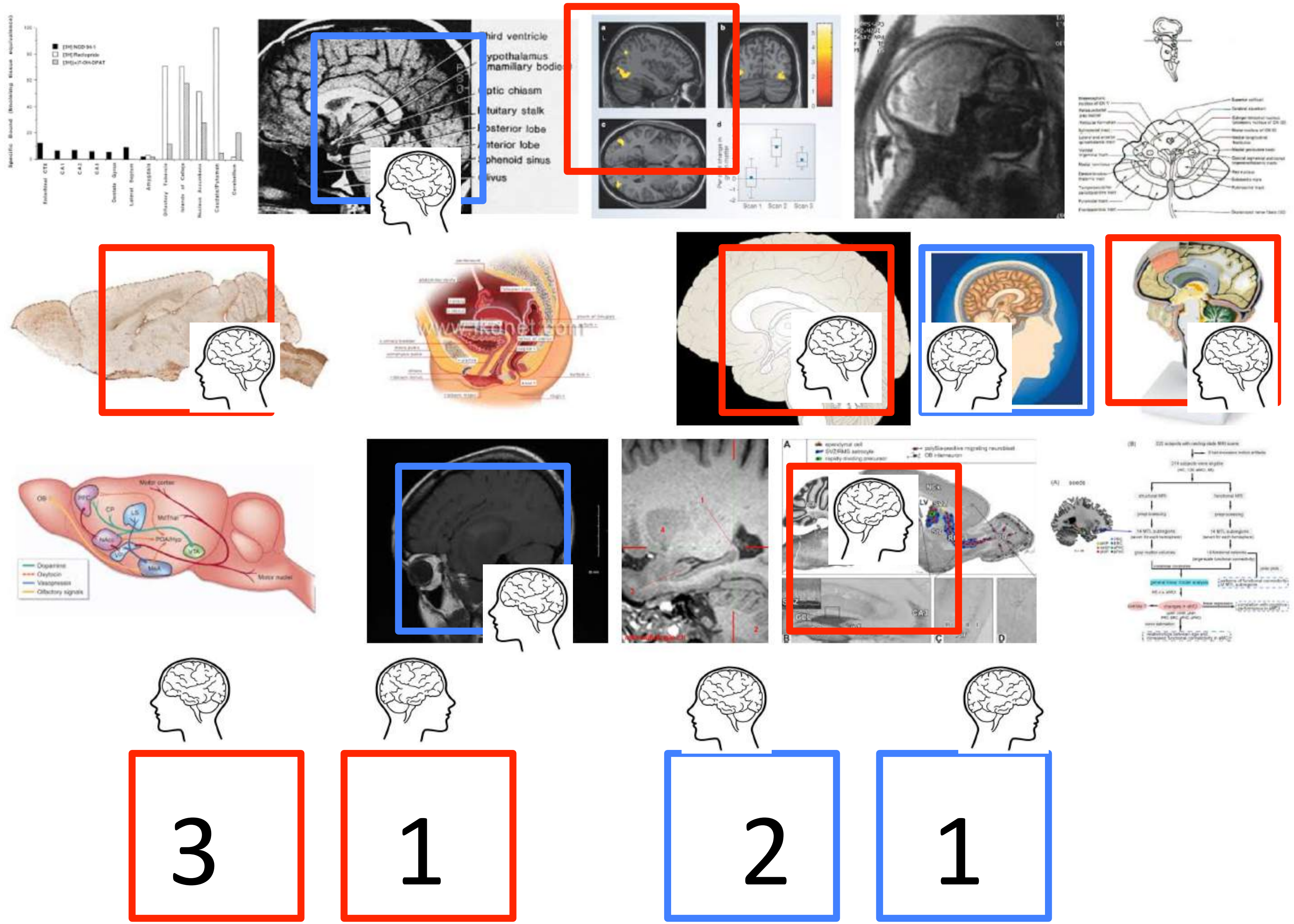
Motor cortex
Dopamine
Oxytocin
Vasopressin
Olfactory signals
Motor nuclei
Scan 1
Scan 2
Scan 3
3
1
2
1

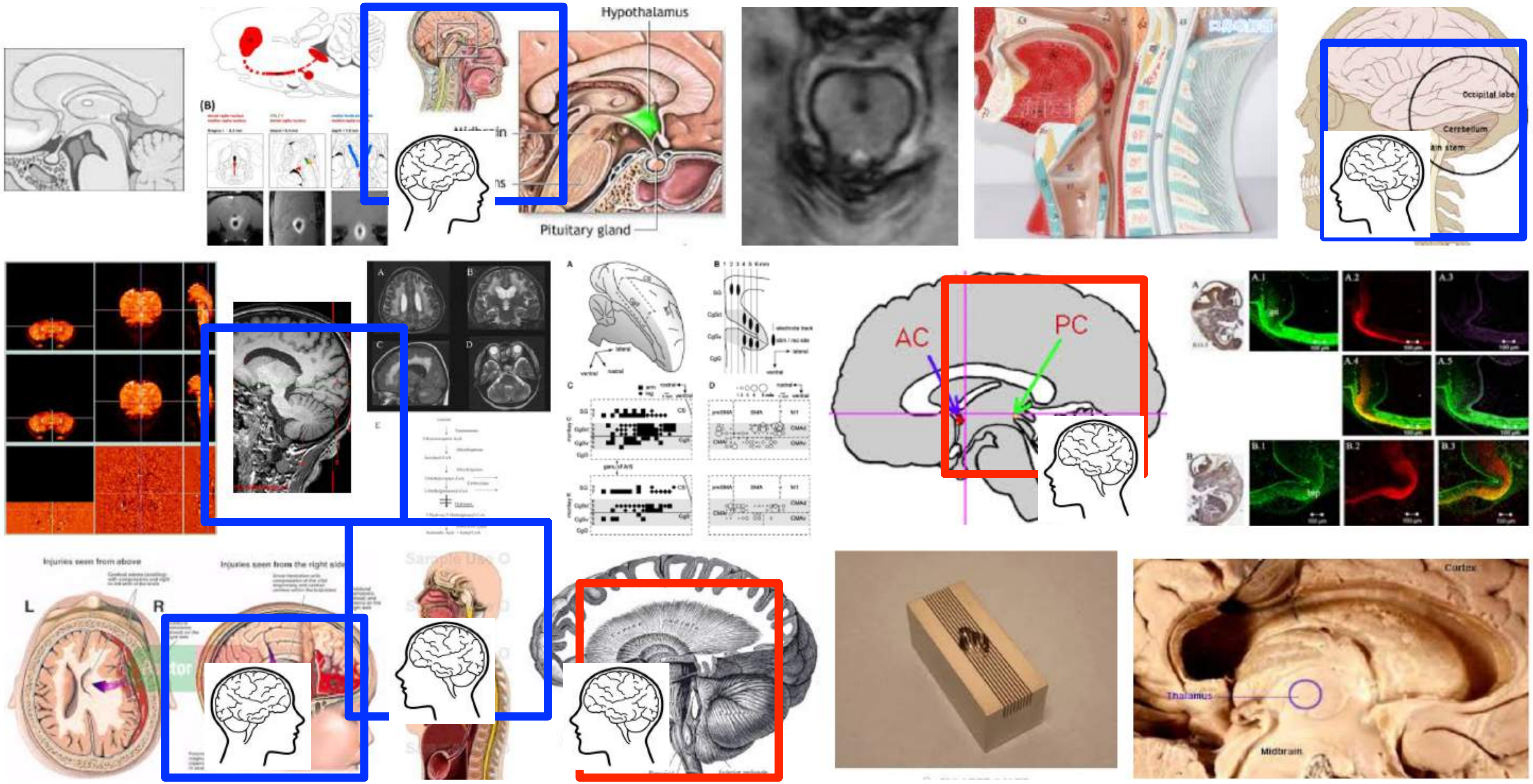
Hypothalamus
Pituitary gland
Occipital lobe
Cerebellum
AC
PC
Injuries seen from above
Injuries seen from the right side
L
R
Cortex
Thalamus
Midbrain

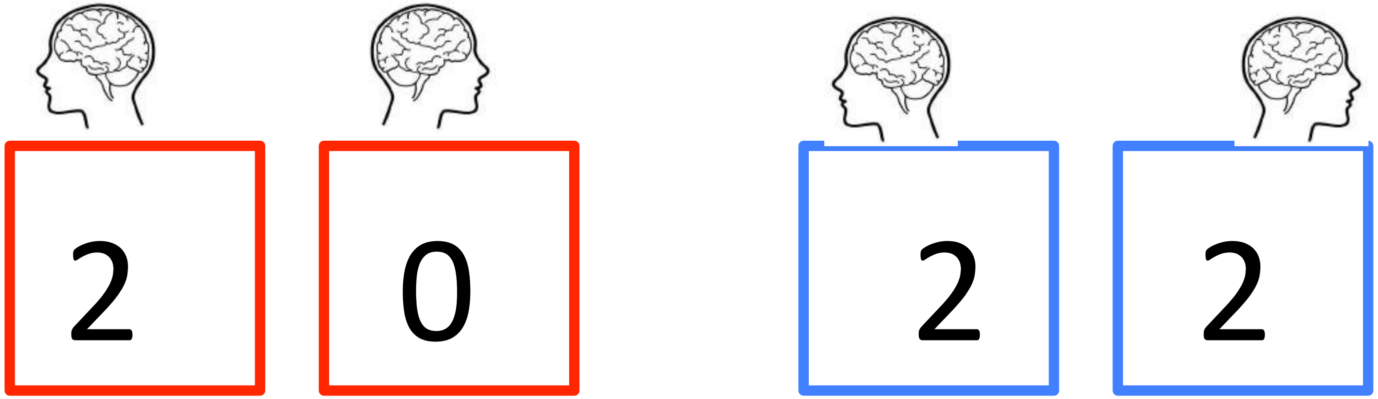

2 0 2 2

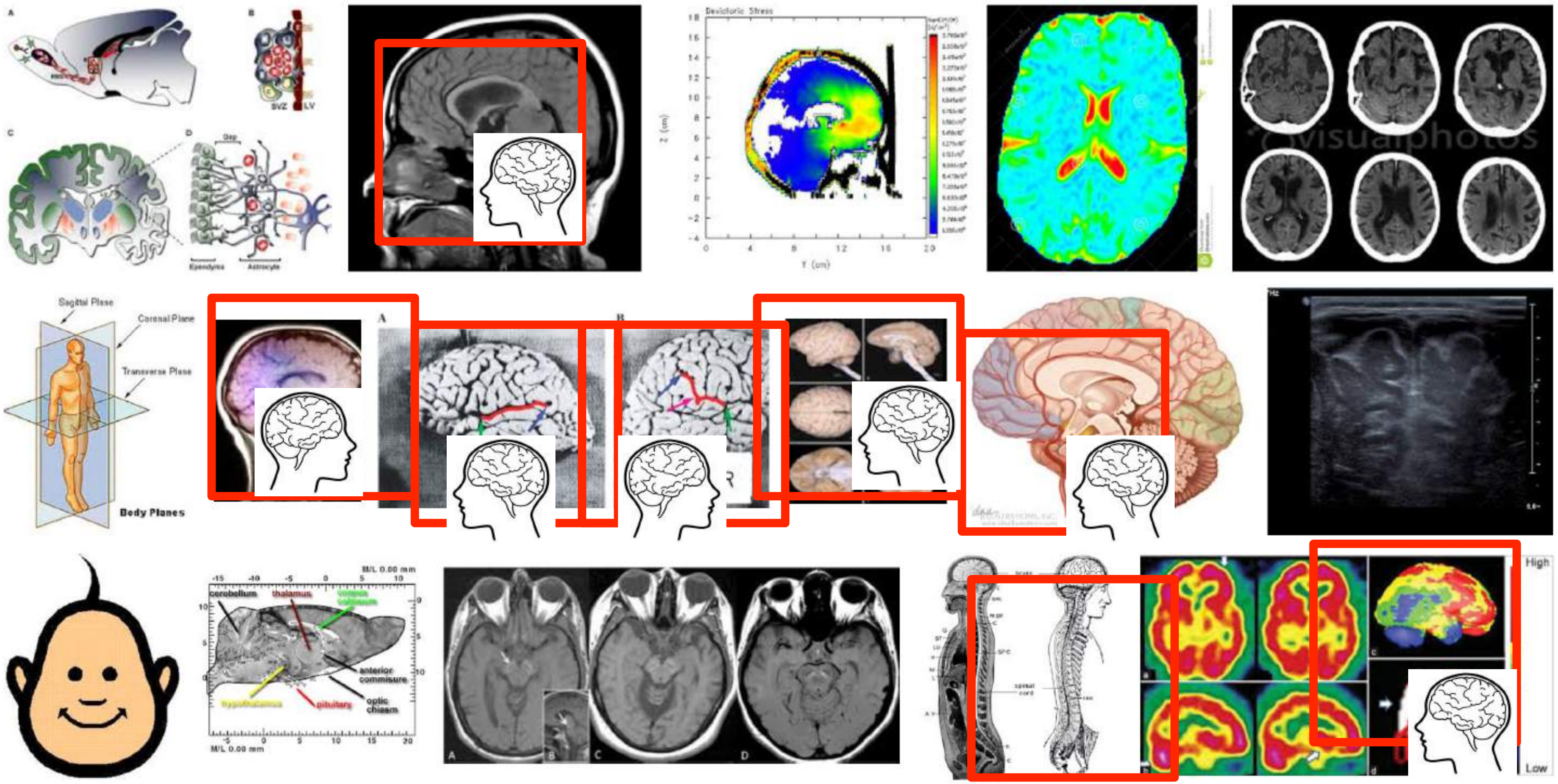
Sagittal Plane
Coronal Plane
Transverse Plane
Body Planes
Deviatoric Stress
cerebellum
thalamus
anterior commisure
optic chiasm
pituitary
High
Low

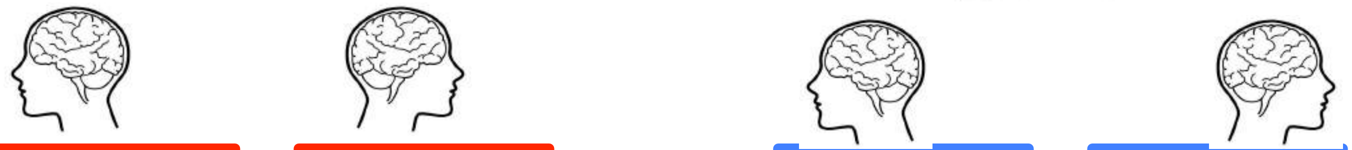

4	1	1	1

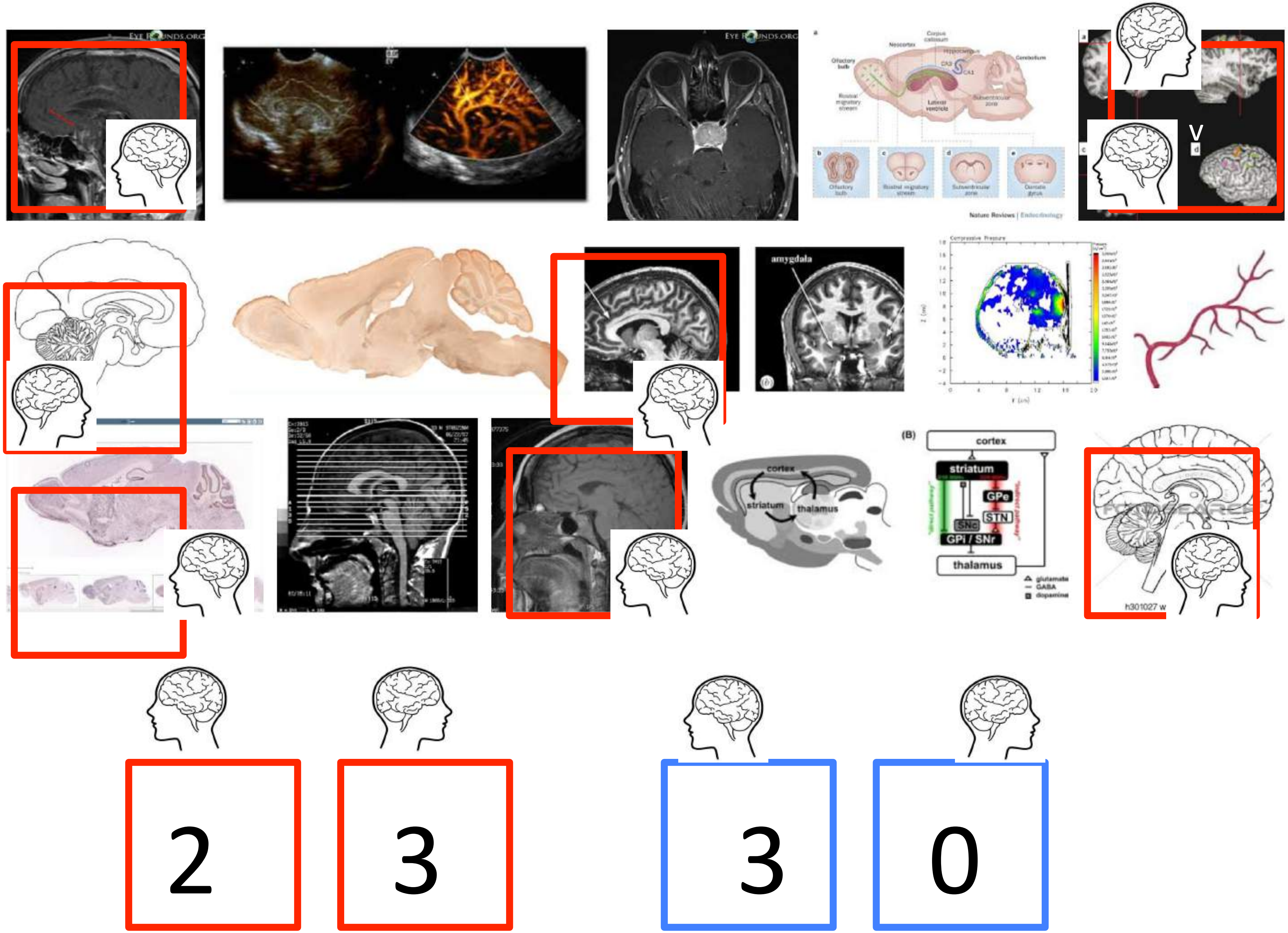

amygdala
cortex
striatum
thalamus
(B)
cortex
striatum
GPe
STN
SNc
GPi / SNr
thalamus
glutamate
GABA
dopamine
h301027 w
V
2
3
3
0

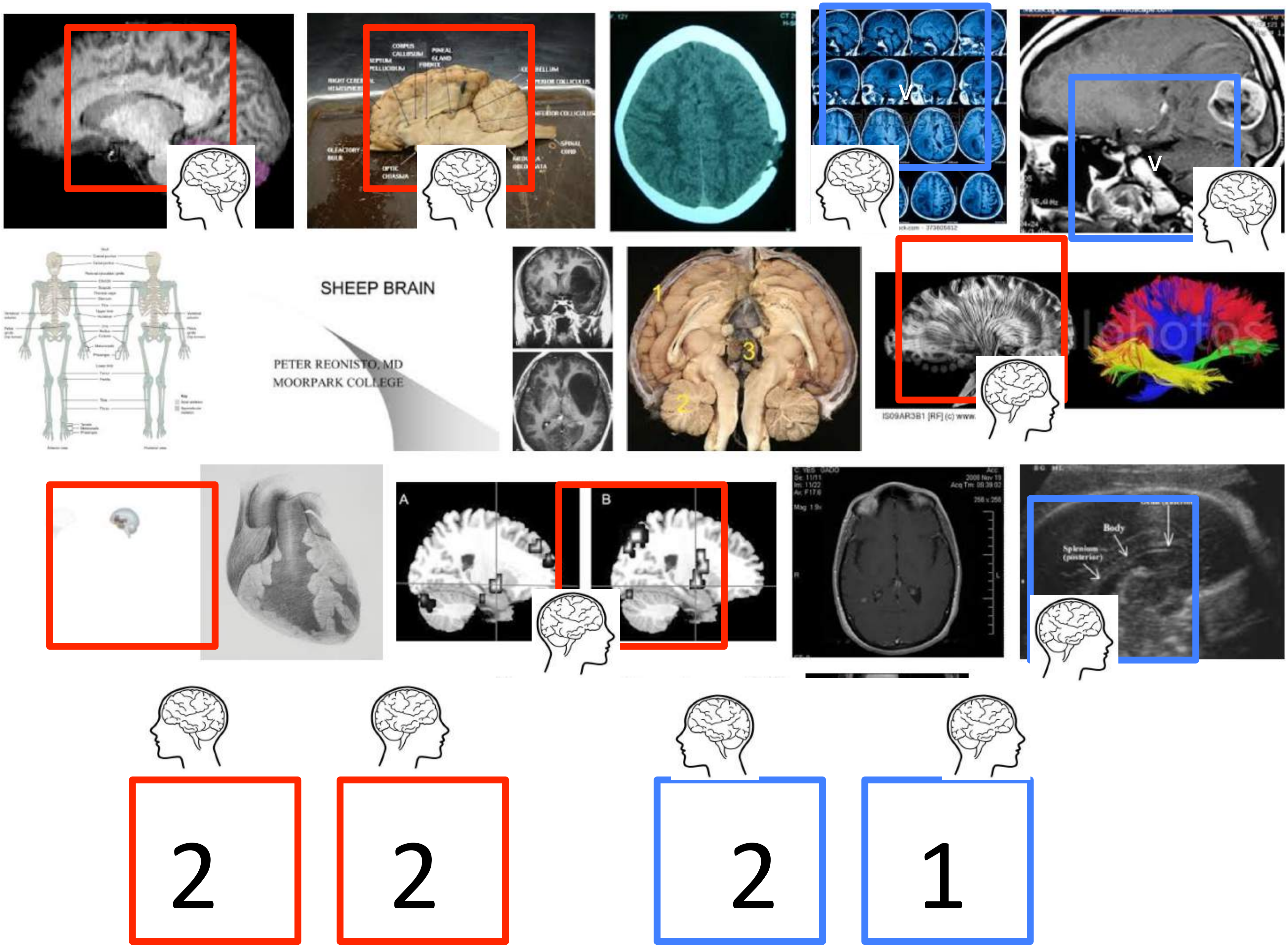
V
V
SHEEP BRAIN
PETER REONISTO, MD
MOORPARK COLLEGE
1
3
2
A
B
Body
Splenium
(posterior)
2
2
2
1

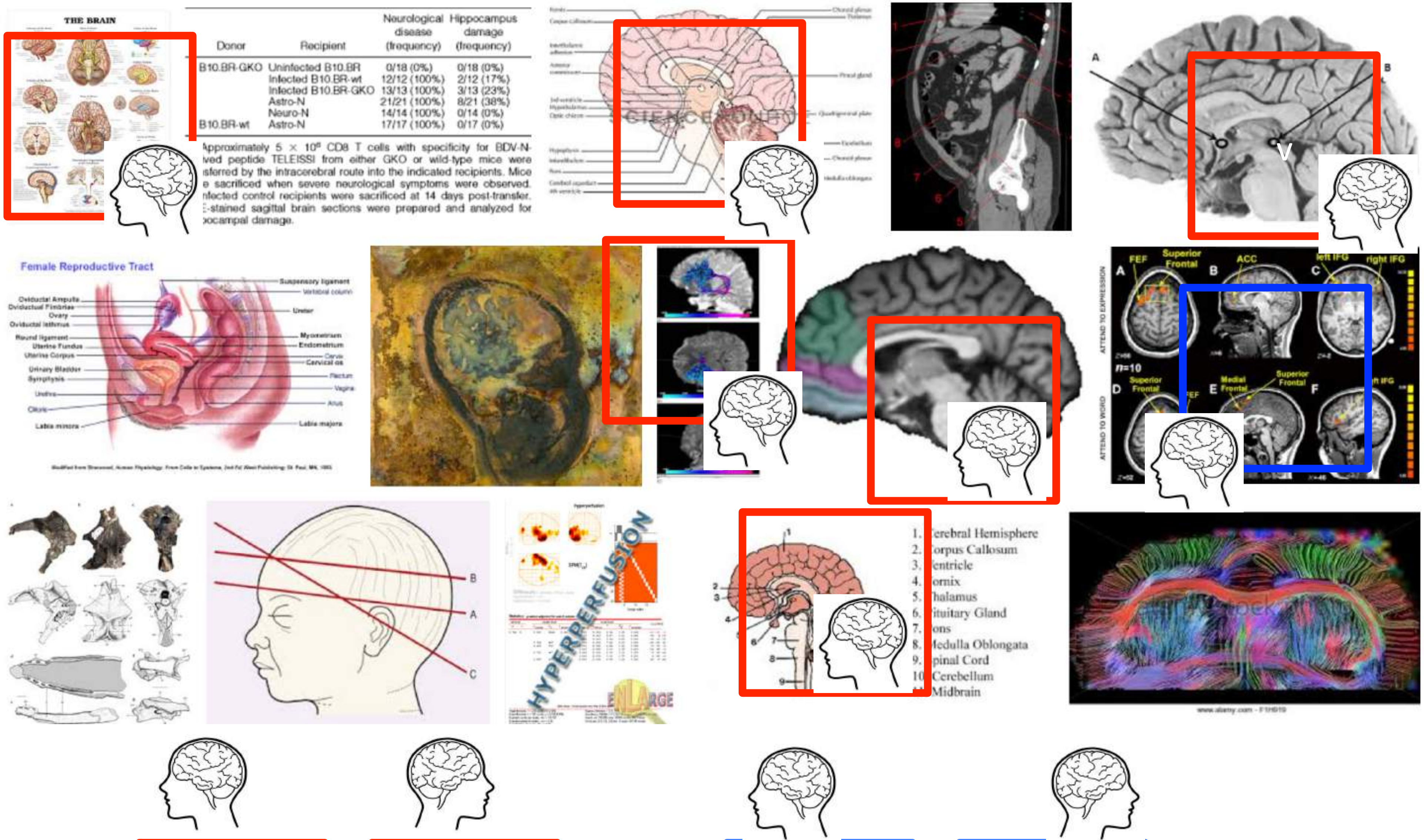

6 0 1 0

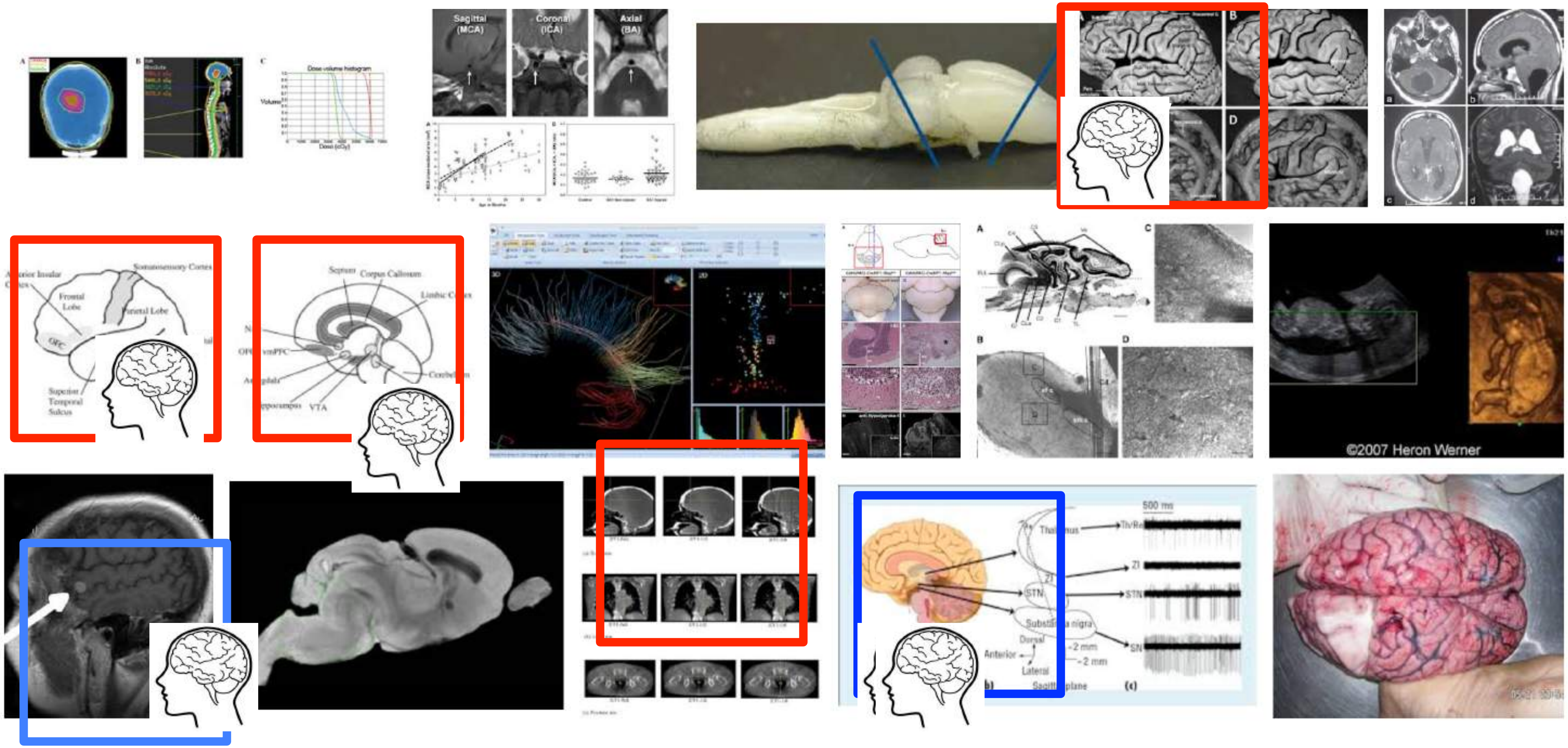

Sagittal (MCA)
Coronal (ICA)
Axial (BA)
Dose volume histogram
Somatosensory Cortex
Frontal Lobe
Parietal Lobe
OFC
Superior Temporal Sulcus
Septum
Corpus Callosum
vmPFC
VTA
©2007 Heron Werner
500 ms
STN
Dorsal
Anterior
Lateral

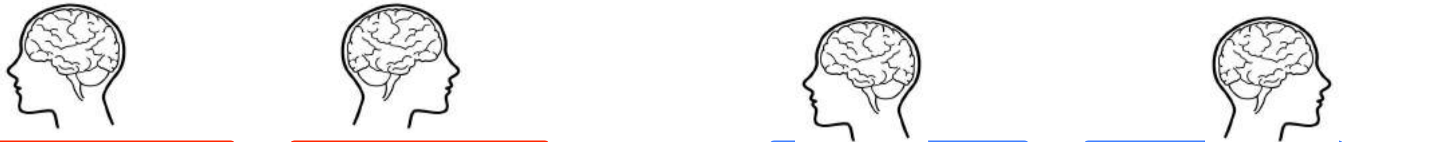

4	0	2	0

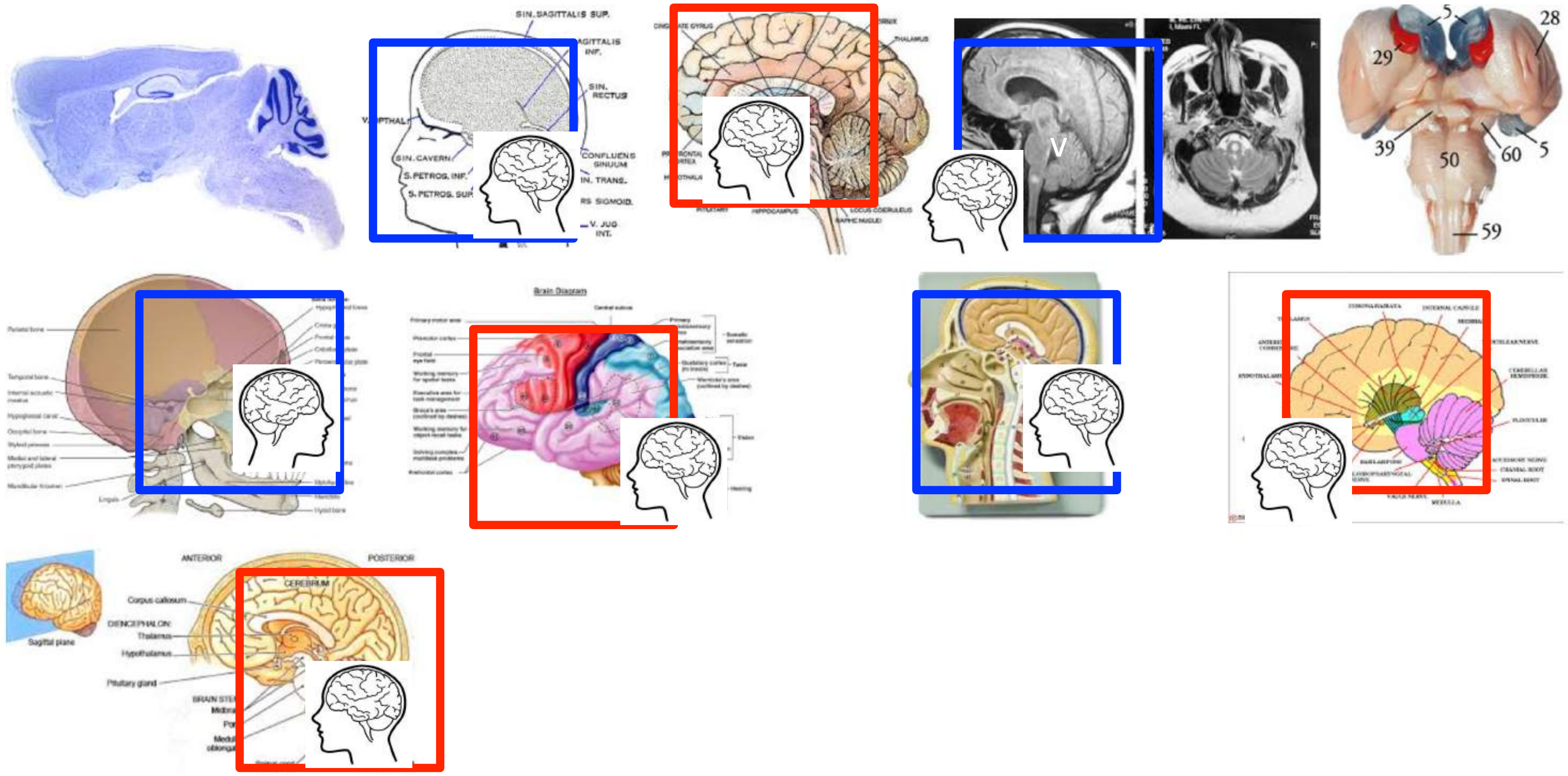
SIN. SAGITTALIS SUP.
SIN. RECTUS
CONFLUENS SINUUM
SIN. CAVERN.
S. PETROS. INF.
S. PETROS. SUP.
Brain Diagram
ANTERIOR
POSTERIOR
CEREBRUM
Corpus callosum
DIENCEPHALON
Thalamus
Hypothalamus
Pituitary gland
Sagittal plane
28
29
5
39
50
60
59
V

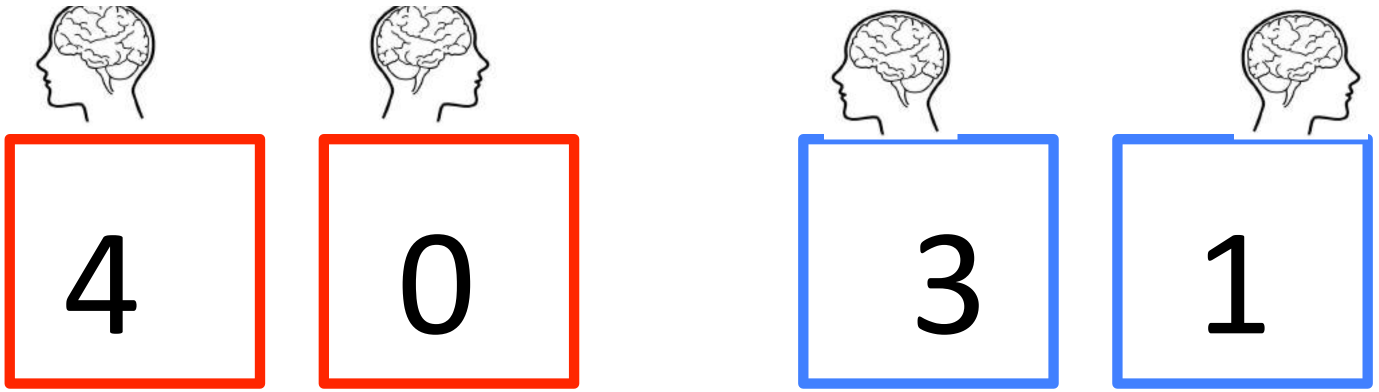
4
0
3
1

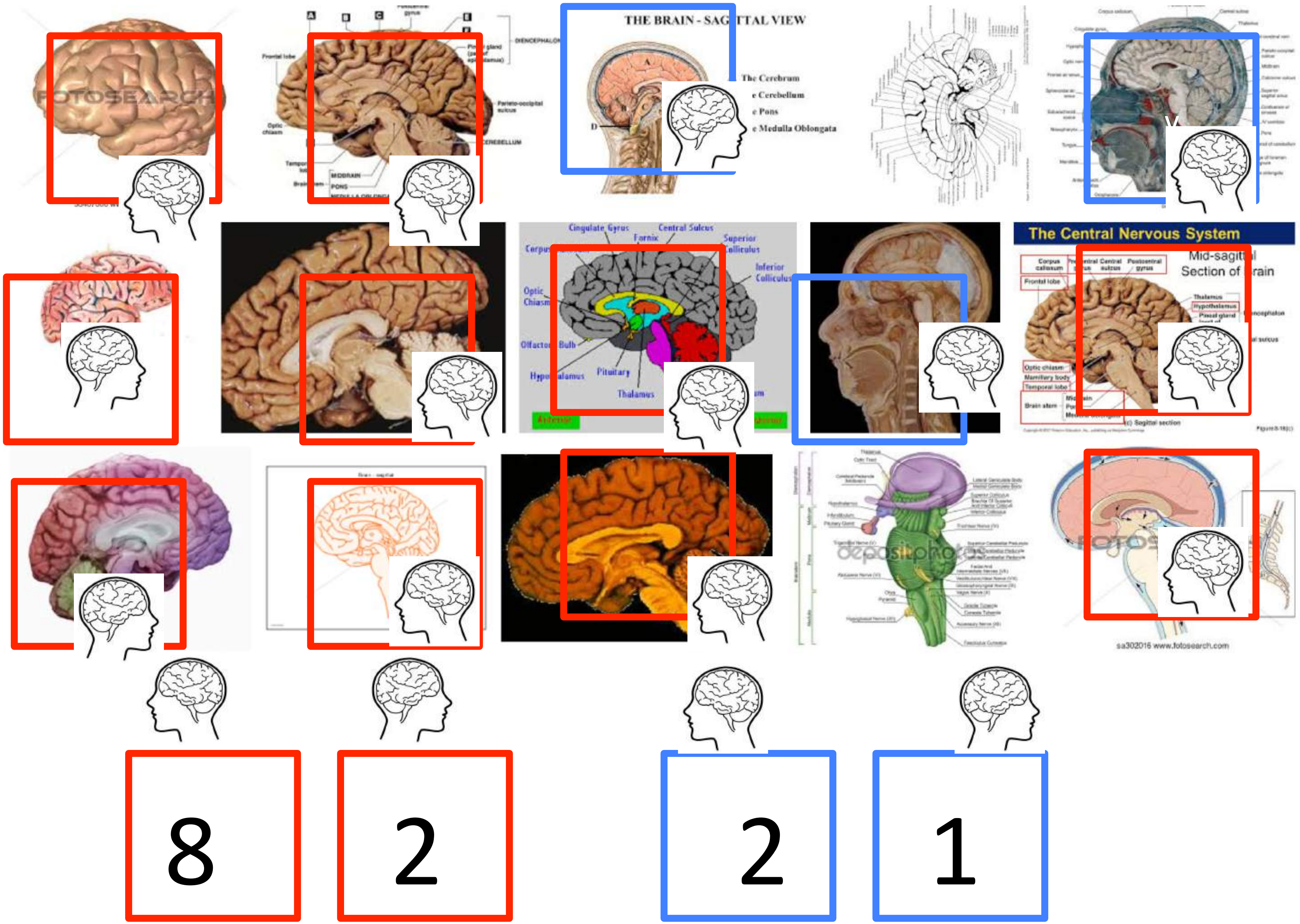
The Cerebrum
The Cerebellum
The Pons
The Medulla Oblongata
Cingulate Gyrus
Central Sulcus
Fornix
Superior Colliculus
Inferior Colliculus
Optic Chiasm
Olfactory Bulb
Pituitary
Thalamus
The Central Nervous System
Section of
sa302016 www.fotosearch.com
8
2
2
1
